# Endothelial HIF**α**/PDGF-B to smooth muscle Beclin1 signaling sustains pathological muscularization in pulmonary hypertension

**DOI:** 10.1172/jci.insight.162449

**Published:** 2024-04-23

**Authors:** Fatima Z. Saddouk, Andrew Kuzemczak, Junichi Saito, Daniel M. Greif

**Affiliations:** 1 Yale Cardiovascular Research Center, Section of Cardiovascular Medicine, Department of Internal Medicine, and; 2 Department of Genetics, Yale University, New Haven, Connecticut, USA.

**Keywords:** Pulmonology, Vascular biology, Autophagy, Cardiovascular disease, Hypoxia

## Abstract

Mechanisms underlying maintenance of pathological vascular hypermuscularization are poorly delineated. Herein, we investigated retention of smooth muscle cells (SMCs) coating normally unmuscularized distal pulmonary arterioles in pulmonary hypertension (PH) mediated by chronic hypoxia with or without Sugen 5416, and reversal of this pathology. With hypoxia in mice or culture, lung endothelial cells (ECs) upregulated hypoxia-inducible factor 1α (HIF1-α) and HIF2-α, which induce platelet-derived growth factor B (PDGF-B), and these factors were reduced to normoxic levels with re-normoxia. Re-normoxia reversed hypoxia-induced pulmonary vascular remodeling, but with EC HIFα overexpression during re-normoxia, pathological changes persisted. Conversely, after establishment of distal muscularization and PH, EC-specific deletion of *Hif1a*, *Hif2a*, or *Pdgfb* induced reversal. In human idiopathic pulmonary artery hypertension, HIF1-α, HIF2-α, PDGF-B, and autophagy-mediating gene products, including Beclin1, were upregulated in pulmonary artery SMCs and/or lung lysates. Furthermore, in mice, hypoxia-induced EC-derived PDGF-B upregulated Beclin1 in distal arteriole SMCs, and after distal muscularization was established, re-normoxia, EC *Pdgfb* deletion, or treatment with STI571 (which inhibits PDGF receptors) downregulated SMC Beclin1 and other autophagy products. Finally, SMC-specific *Becn1* deletion induced apoptosis, reversing distal muscularization and PH mediated by hypoxia with or without Sugen 5416. Thus, chronic hypoxia induction of the HIFα/PDGF-B axis in ECs is required for non–cell-autonomous Beclin1-mediated survival of pathological distal arteriole SMCs.

## Abstract 2

In pulmonary hypertension, endothelial HIF&alpha; promotes platelet-derived growth factor B signaling to smooth muscle Beclin1, which is required for retention of pathological distal arteriole muscularization.

## Graphical abstract

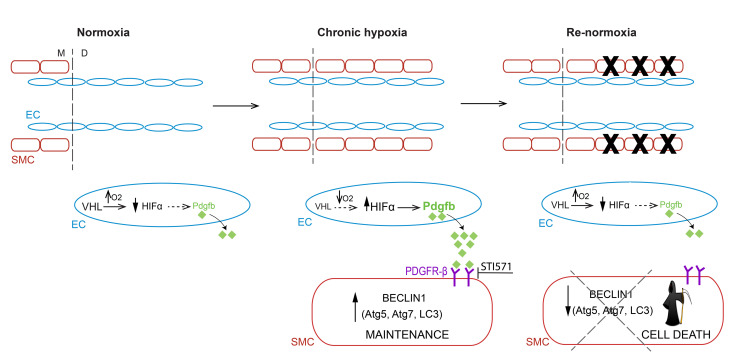

## Introduction

Chronic cardiovascular diseases, such as atherosclerosis, arterial restenosis, and pulmonary hypertension (PH), are characterized by excess and aberrant smooth muscle cells (SMCs). The processes leading to this hypermuscularization are highly studied, whereas once pathological SMCs are established, mechanisms — both cell autonomous and non–cell autonomous — responsible for their maintenance are not well understood. In pulmonary artery hypertension (PAH; classified by the World Health Organization as Group 1 PH), reduced compliance of the pulmonary vasculature predicts mortality (1), and the distal extension of SMCs to normally unmuscularized pulmonary arterioles reduces compliance. PAH treatments primarily induce vascular dilation, but generally do not ameliorate hypermuscularization, and unfortunately, this disease remains highly morbid and lethal, with approximately 50% of patients dying within 7 years of diagnosis (2). From a therapeutic standpoint, it is critical to elucidate how to reverse distal arteriole muscularization because when symptomatic patients initially present for clinical evaluation, invariably their distal pulmonary arterioles are muscularized. Thus, a fundamental question arises: what cellular processes and molecular pathways can be manipulated to reverse pathological remodeling?

High-altitude residents and acclimatized climbers are at risk for developing muscularized distal pulmonary arterioles and PH due to hypobaric hypoxia, and relocation to lower altitudes often normalizes pulmonary artery (PA) pressure (3–6). This exposure to increased oxygen levels at lower altitudes is thought to also result in regression of distal arteriole muscularization (5, 6). In rats, re-exposure to normoxia following hypoxia induces vascular cell apoptosis and reverses pathological vascular remodeling (7, 8). Apoptosis resistance of PA SMCs (PASMCs) is a hallmark of PH, and during re-oxygenation, there is an increase in apoptosis that contributes to the regression of vascular remodeling (8). However, the cellular events underlying reversal and the molecular mechanisms regulating this process are poorly understood.

Hypoxia-inducible factors (HIFs) modulate gene transcription in an oxygen-dependent manner and are key players in inducing pulmonary vascular remodeling and PH (9–11). HIFs are heterodimers of HIF1-β and either HIF1-α or HIF2-α. Mice heterozygous for the global null allele *Hif1a* or *Hif2a* or with SMC-specific deletion of *Hif1a* via *Myh11-CreER^T2^* *Hif1a^fl/fl^* have attenuated hypoxia-induced PH and lung vascular disease (12–14). Similarly, *Hif2a^fl/fl^* mice with *Hif2a* deletion conditionally in endothelial cells (ECs) with *Tie2-CreER^T2^* or constitutively in pulmonary ECs with *L1-Cre* are protected against PH (15, 16). However, in established disease, the roles of HIFs in retention of pathological SMC remodeling under chronic hypoxia and its reversal are not well studied.

Hypoxia induces EC expression of diverse growth factors and agonists that non–cell autonomously regulate the biology of PASMCs and are implicated in PH and pulmonary vascular remodeling (17–20). We have previously shown that a hypoxia-induced EC HIF1-α to platelet-derived growth factor B (PDGF-B) pathway regulates PASMC proliferation and de-differentiation (10). Yet, whether ECs play a key role in sustaining distal arteriole muscularization and its reversal has not been investigated. PDGF-B induces autophagy in SMCs (21, 22). Beclin1 is a critical initiator of the autophagic pathway, and Beclin1 expression is upregulated in hypoxia-induced PH and is positively related to autophagy in PH (23, 24). Enhanced migration and proliferation of *Becn1^+/–^* lung ECs is dependent on HIF2-α (25). However, the pathophysiological functions of Beclin1 in PH and vascular remodeling are not well studied.

In the current study, we demonstrate that ECs are non–cell autonomously essential for the retention of established hypoxia-induced distal arteriole SMCs. SMC HIFα is not required for sustaining pathological distal arteriole muscularization, whereas deletion of *Hif1a* or *Hif2a* in ECs following hypoxia-induced distal arteriole muscularization leads to reversal of this muscularization despite continued hypoxia. HIF1-α and HIF2-α regulate *Pdgfb* expression under chronic hypoxia, and *Pdgfb* deletion in ECs following hypoxia-induced distal arteriole muscularization reverses this pathology, highlighting the importance of EC-SMC interactions in the retention of pathological hypermuscularization. Finally, we show the non–cell-autonomous effects of EC-derived PDGF-B induction of PASMC Beclin1 and that the latter is essential for the retention of distal arteriole muscularization by augmenting autophagy and inhibiting apoptosis of SMCs.

## Results

### Re-normoxia reverses hypoxia-induced distal arteriole muscularization and PH.

The distal extension of smooth muscle coverage to normally unmuscularized distal pulmonary arterioles is a hallmark of human and rodent PH (26–28). As in our prior studies, we focus on specific pulmonary arteriole beds adjacent to identified airway branches (left bronchus–first lateral secondary branch–first anterior branch or left bronchus–first medial branch) in the adult mouse lung (10, 27–29) (Supplemental Figure 1A; supplemental material available online with this article; https://doi.org/10.1172/jci.insight.162449DS1). Muscularized PAs and arterioles are identified by their continuous layer of α-smooth muscle actin^+^ (SMA)^+^ SMCs, whereas pulmonary veins have a coating of SMCs that form a relatively loose mesh around the vessel (30), and *Bmx-CreER^T2^* marks ECs of arteries and arterioles but not venous ECs (Supplemental Figure 1B) (31). Distal arterioles in these beds are unmuscularized normally, but with hypoxia exposure undergo a stereotyped process of muscularization, resulting in PH and right ventricle (RV) hypertrophy (RVH) (27, 28). Herein, we investigate these specific arteriole beds to assess the reversal of hypoxia-induced vascular remodeling upon re-normoxia.

**Figure 1:**
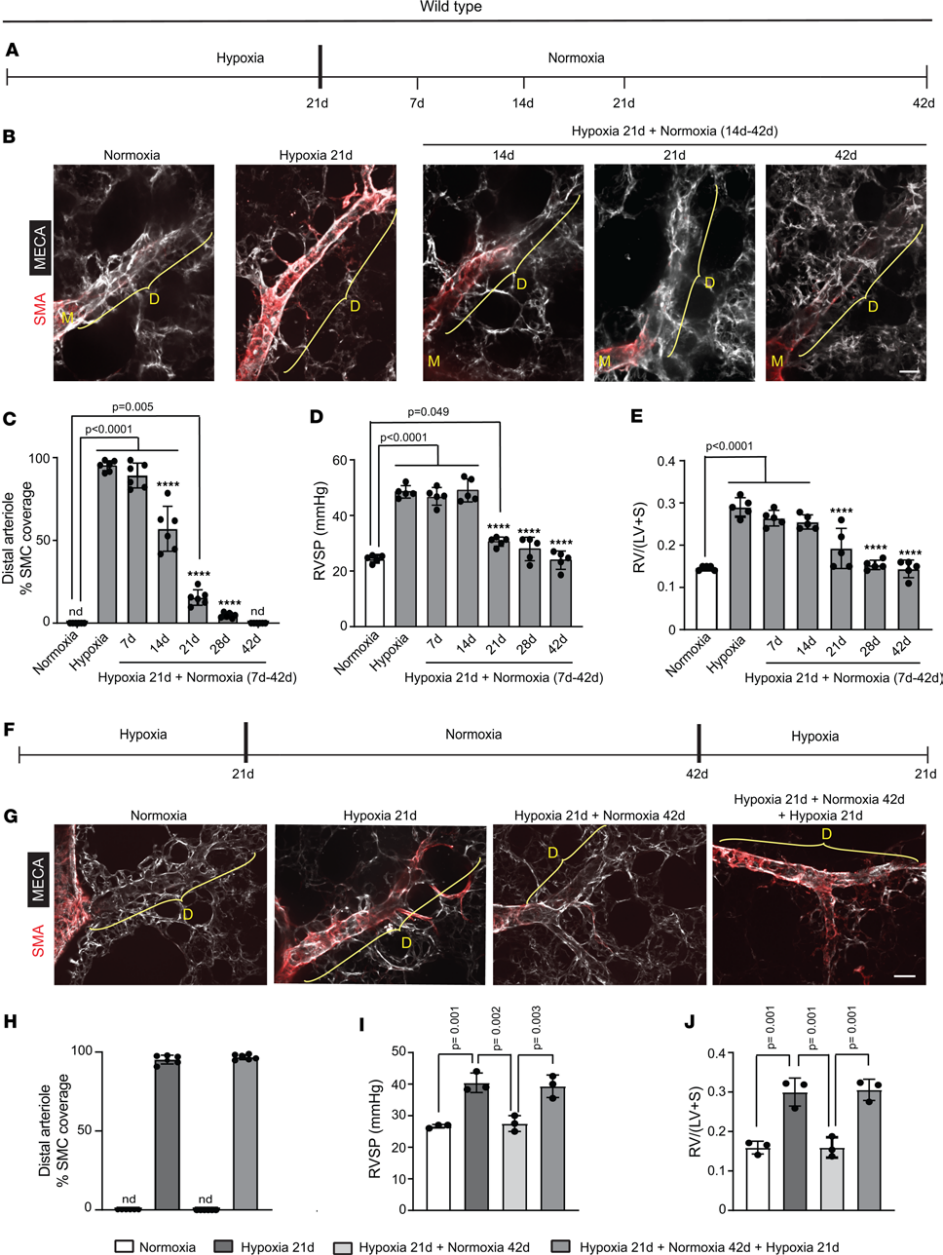
Reversal of distal arteriole muscularization with re-normoxia following hypoxia. (**A**) Experimental strategy for **B**–**E**. (**B**–**E**) Wild-type mice were maintained in normoxia or were exposed to hypoxia for 21 days and either analyzed at that point or following re-exposure to normoxia for 7–42 days as indicated. (**B**) Vibratome lung sections were stained for SMA and the EC marker MECA-32. M and D, middle and distal arterioles are indicated, respectively. (**C**) Percentage of distal arterioles covered by SMCs. (**D**) RVSP (equivalent to PA systolic pressure). (**E**) The RV weight ratio (weight of the RV divided by the sum of left ventricle [LV] and septum [S] weight) were measured. *n* = 5–6 mice (3 males, 2–3 females) per experimental group and 3 arterioles per mouse. nd, not detected. ****P < 0.0001 vs. hypoxia by multifactor ANOVA with Tukey’s multiple-comparison test. (**F**) Experimental strategy for **G**–**J**. (**G**–**J**) Wild-type mice were exposed to (i) normoxia, (ii) hypoxia for 21 days, (iii) hypoxia for 21 days followed by normoxia for 42 days, or (iv) hypoxia for 21 days, normoxia for 42 days, and hypoxia again for 21 days. (**G**) Vibratome lung sections were stained for SMA and MECA-32. (**H**–**J**) Percentages of distal arteriole covered by SMCs, RVSP, and RV/(LV + S) were measured. *n* = 3 mice (1 male, 2 females). Significance evaluated by multifactor ANOVA with Tukey’s multiple-comparison test. nd, not detected. Scale bars: 20 μm.

To evaluate whether hypoxia-induced distal arteriole muscularization, RVH, and/or PH in mice are reversible, *Bmx-CreER^T2^* *ROSA26R^mTmG/mTmG^* or wild-type mice were exposed to hypoxia (FiO_2_ 10%) for 21 days and then transferred to re-normoxia (FiO_2_ 21%) for up to 28 or 42 days, respectively (Figure 1A and Supplemental Figure 1C). Distal muscularization started to regress by re-normoxia day 14 (Figure 1, B and C, and Supplemental Figure 1D) and by day 21 there was a significant reduction in the RV systolic pressure (RVSP; equivalent to PA systolic pressure) and weight ratio of RV to the sum of the left ventricle (LV) and septum (S), which is a measure of RVH (Figure 1, D and E). By re-normoxia day 42, the 3 parameters measured — distal muscularization, RVSP, and RV weight ratio — were normalized and did not differ significantly from the basal normoxic state. We then investigated whether hypoxia-induced distal muscularization can be recapitulated by exposing these recovered mice to hypoxia once again. Mice were exposed to 21 days of hypoxia followed by normoxia for 42 days and then re-exposed to hypoxia for 21 days (Figure 1F). Mice re-exposed to hypoxia developed distal arteriole muscularization, PH, and RVH, at levels equivalent to the initial 21-day hypoxic mice (Figure 1, G–J).

Given that re-normoxia leads to reversal of hypoxia-induced distal muscularization in mice, we next evaluated whether distal arteriole SMCs downregulate SMA expression and change fate or undergo cell death during the re-normoxia phase. *Acta2-CreER^T2^ ROSA26R^mTmG/+^* mice were induced with tamoxifen, rested for 5 days, and then exposed to (i) normoxia for an additional 32 days; (ii) hypoxia for 21 days; or (iii) hypoxia for 21 days, followed by re-normoxia for 42 days (Supplemental Figure 2A). In agreement with our prior results (27), on hypoxia day 21, the vast majority of distal arteriole SMCs were GFP^+^, indicating that they were derived from preexisting SMCs (Supplemental Figure 2B). With re-normoxia, only very rare distal arteriole SMA^+^ cells persisted and these cells were invariably GFP^+^, and essentially all GFP^+^ lung cells were SMA^+^ (Supplemental Figure 2B). Thus, distal arteriole SMCs do not undergo a fate change (e.g., de-differentiation or trans-differentiation) to an SMA^–^ cell type with re-normoxia. Furthermore, on day 7 of re-normoxia, 7% ± 1% of distal arteriole SMCs were TUNEL^+^ (Supplemental Figure 2, C and D), indicating that similar to prior studies in rats (8), exposure to re-normoxia induces programmed cell death of SMCs coating small vessels.

### SMC HIFα is neither necessary nor sufficient for retention of distal arteriole muscularization.

**Figure 2:**
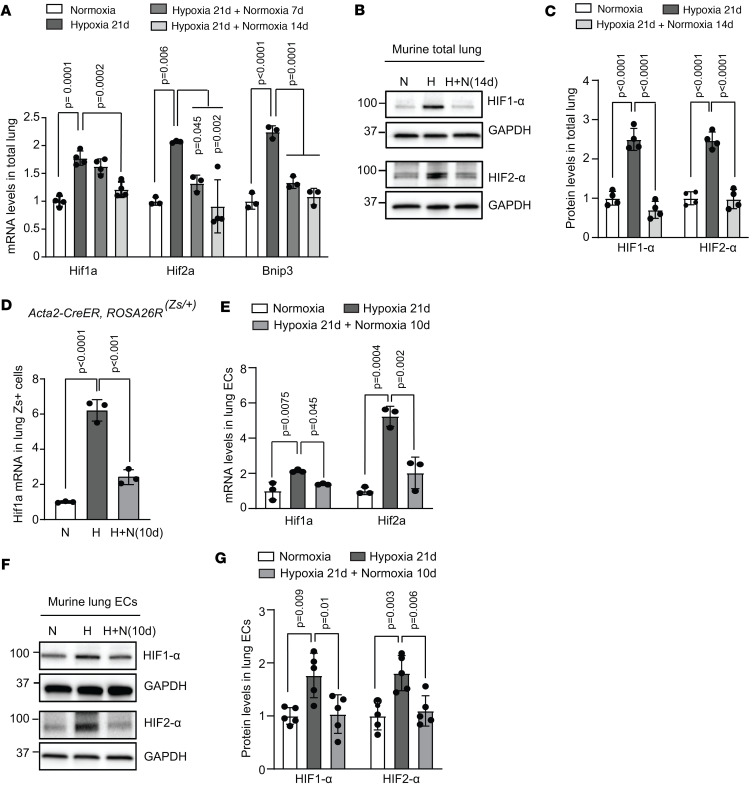
HIFα is upregulated with hypoxia and downregulated with re-normoxia. (**A**) Wild-type mice were exposed to either normoxia or hypoxia for 21 days with or without subsequent re-normoxia for 7 or 14 days. Lung lysates were analyzed by qRT-PCR to assess *Hif1a*, *Hif2a*, and *Bnip3* levels. *n* = 3–4 mice (2 males, 1–2 females) per experimental group. (**B** and **C**) Wild-type mice were exposed to normoxia or hypoxia for 21 days and then were or were not subjected to normoxia for 14 days. Whole-lung lysates were analyzed by Western blot for HIF1-α, HIF2-α, and GAPDH protein (**B**), with densitometry relative to GAPDH and normalized to normoxia (**C**). *n* = 4 mice (2 males, 2 females) per experimental group. (**D**) *Acta2-CreER^T2^* *ROSA26R^Zs/+^* mice were induced with tamoxifen (1 mg/day for 5 days), rested for 5 days, exposed to hypoxia for 21 days, and then re-normoxia for 10 days. Zs^+^ cells were isolated by FACS, and *Hif1a* expression levels were measured by qRT-PCR. *n* = 3 mice (1 male, 2 females) per experimental group. (**E**–**G**) Wild-type mice were exposed to normoxia or hypoxia for 21 days followed by normoxia for 10 days. (**E**) Lung CD31^+^CD45^–^ ECs were isolated by FACS, and transcript levels of *Hif1a* and *Hif2a* were measured by qRT-PCR. *n* = 3 (1 male, 2 females) per experimental group. (**F** and **G**) Lung ECs were isolated with anti-CD31–coated beads. Western blot analysis of EC lysates for HIF1-α, HIF2-α, and GAPDH protein are shown (**F**) with densitometry relative to GAPDH and normalized to normoxia (**G**). *n* = 5 mice (2 males, 3 females) per experimental group. Multifactor ANOVA with Tukey’s multiple-comparison test was used (**A**, **C**–**E**, and **G**). N, normoxia; H, hypoxia; H+N, hypoxia followed by normoxia.

HIFs are heterodimers of HIF1-β and either HIF1-α or HIF2-α. Herein, mRNA and protein levels of HIF1-α, HIF2-α, and their transcriptional target BCL2-interacting protein 3 (Bnip3) were found to be upregulated in whole-lung lysates of mice exposed to hypoxia for 21 days and then decreased when mice were returned to normoxia for 7 or 14 days (Figure 2, A–C). In PASMCs, HIF1-α is the main isoform, as HIF2-α is not readily detectable (32), and *Hif1a* deletion in SMCs protects mice against hypoxia-induced distal pulmonary arteriole muscularization and PH (10, 14). To investigate the dynamics of SMC HIF1-α levels in the context of PH reversal, ZsGreen1^+^ (Zs^+^) cells were isolated by fluorescence-activated cell sorting (FACS) from the lungs of tamoxifen-induced *Acta2-CreER^T2^ ROSA26R^Zs/+^* mice exposed to normoxia, hypoxia, or hypoxia followed by 10 days of re-normoxia. The marked hypoxia-induced HIF1-α levels in Zs^+^ SMCs were mitigated upon re-normoxia (Figure 2D). Because HIFα in ECs is critical for developing distal pulmonary arteriole muscularization and PH in response to hypoxia (10, 15, 33), we next evaluated the role of EC HIFα in sustaining these pathological changes with chronic hypoxia. Lung ECs were isolated by FACS from mice exposed to normoxia, hypoxia, or hypoxia followed by 10 days of re-normoxia, and HIF1-α and HIF2-α levels increased with hypoxia and then decreased with re-normoxia (Figure 2, E–G).

**Figure 3:**
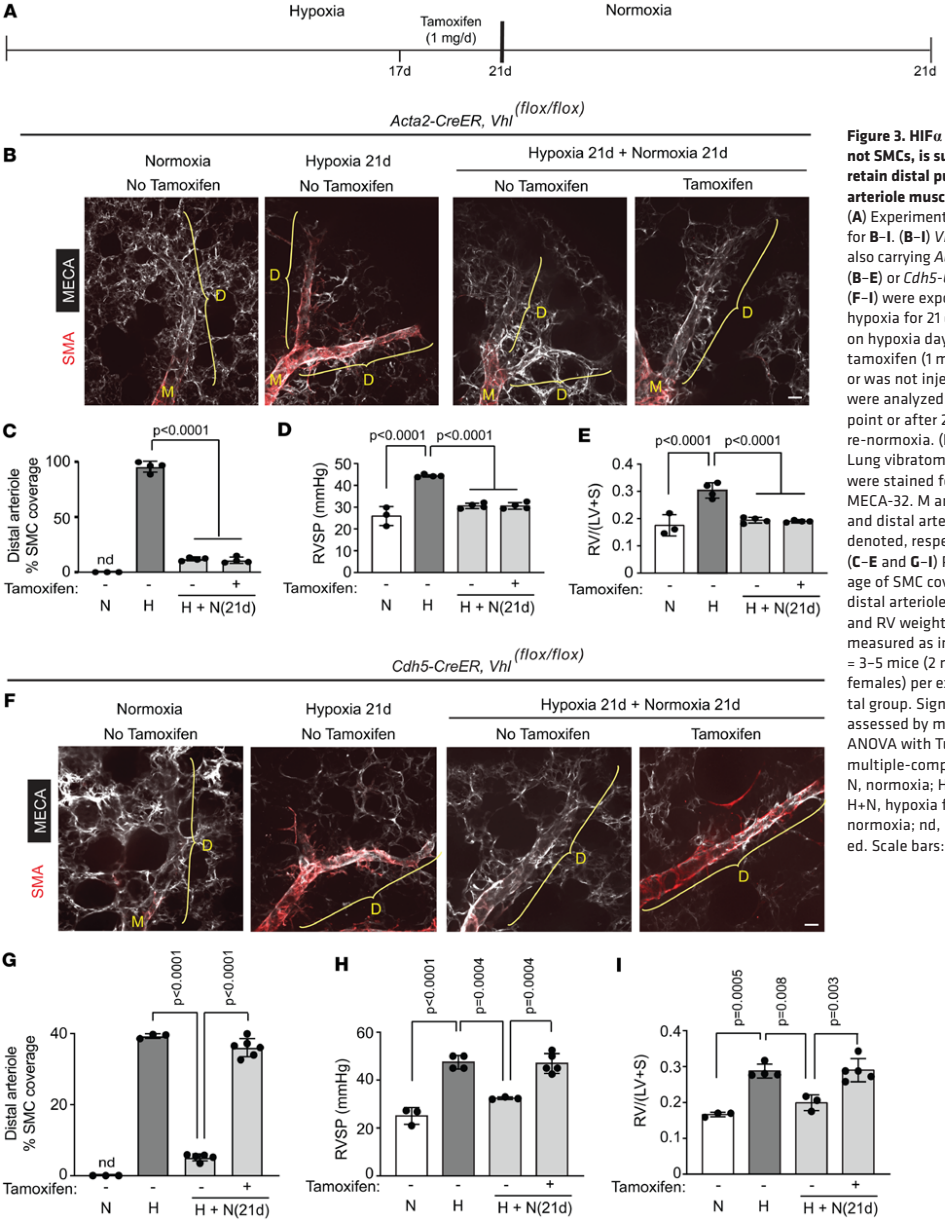
HIFα in ECs, but not SMCs, is sufficient to retain distal pulmonary arteriole muscularization. (**A**) Experimental strategy for **B**–**I**. (**B**–**I**) *Vhl^fl/fl^* mice also carrying *Acta-CreER^T2^* (**B**–**E**) or *Cdh5-CreER^T2^* (**F**–**I**) were exposed to hypoxia for 21 days and on hypoxia days 17–21, tamoxifen (1 mg/day) was or was not injected. Mice were analyzed at this time point or after 21 days of re-normoxia. (**B** and **F**) Lung vibratome sections were stained for SMA and MECA-32. M and D, middle and distal arterioles are denoted, respectively. (**C**–**E** and **G**–**I**) Percentage of SMC coverage of distal arterioles, RVSP, and RV weight ratio were measured as indicated. *n* = 3–5 mice (2 males, 1–3 females) per experimental group. Significance assessed by multifactor ANOVA with Tukey’s multiple-comparison test. N, normoxia; H, hypoxia; H+N, hypoxia followed by normoxia; nd, not detected. Scale bars: 20 μm.

Based on the dynamics of HIFα expression and distal pulmonary arteriole muscularization, we initially postulated that SMC HIF1-α may play a key role in sustaining distal muscularization and PH under hypoxic conditions. To investigate this hypothesis, *Acta2-CreER^T2^ Hif1a^fl/fl^* mice were exposed to hypoxia for 49 days and tamoxifen (1 mg/day) was or was not administered on days 17–21 to delete *Hif1a* (Supplemental Figure 3A). Surprisingly, tamoxifen-induced *Hif1a* deletion in SMCs had no effect on distal muscularization, PH, or RVH in hypoxic mice (Supplemental Figure 3, B–D). We next employed a complementary strategy to upregulate HIFα in SMCs under normoxic conditions. HIFα is kept at a low level in normoxia by proline hydroxylation, which facilitates binding to the von Hippel–Lindau–E3 (VHL-E3) ubiquitin ligase complex, resulting in protein ubiquitination and degradation (34, 35). In contrast, HIFα accumulates with hypoxia because oxygen is not available as a substrate for proline hydroxylation. Under normoxic conditions, *Acta2-CreER^T2^* or *Acta2-CreER^T2^ ROSA26R^Zs/+^* mice also carrying *Vhl^fl/fl^* or wild type for *Vhl* were induced with tamoxifen and analyzed 5 days later to assess deletion efficiency (Supplemental Figure 4A). Lung Zs^+^ SMCs were isolated by FACS, and quantitative real-time reverse transcription PCR (qRT-PCR) showed an approximately 70% deletion efficiency of *Vhl* and 3-fold upregulation of *Hif1a* (Supplemental Figure 4B). Similarly, at the protein level, tamoxifen treatment of *Acta2-CreER^T2^ Vhl^fl/fl^* mice markedly reduced VHL in SMCs and upregulated HIF1-α in lung lysates (Supplemental Figure 4, C–E). Next, *Acta2-CreER^T2^* *Vhl^fl/fl^* mice were treated with tamoxifen and rested for 37 days in normoxia, which does not induce distal muscularization, PH, or RVH (Supplemental Figure 4, F–I). In a subsequent set of experiments, *Acta2-CreER^T2^ Vhl^fl/fl^* mice were exposed to hypoxia for 21 days with or without daily tamoxifen on days 17–21 and then returned to normoxia for 21 days and analyzed (Figure 3, A and B). Tamoxifen-induced *Vhl* deletion in SMCs did not affect reversal of distal muscularization, PH, and RVH with re-normoxia (Figure 3, C–E). Taken together, these results refute our initial hypothesis and instead indicate that SMC HIF1-α is not required for the retention of hypoxia-induced distal muscularization and PH.

### EC HIFα is sufficient for sustaining hypoxia-induced distal muscularization and PH.

In stark contrast to *Vhl* deletion in SMCs, we confirmed previous results (10) showing that normoxic *Cdh5-CreER^T2^ Vhl^fl/fl^* mice, which have lung ECs with reduced VHL and elevated HIF1-α and HIF2-α (Supplemental Figure 5, A–C), develop distal arteriole muscularization and extended these findings to demonstrate that these mice develop PH and RVH (Supplemental Figure 5, D–F). Next, *Cdh5-CreER^T2^ Vhl^fl/fl^* mice were exposed to hypoxia for 21 days with or without tamoxifen (1 mg/day) administration on days 17–21 and then either analyzed on hypoxia day 21 as a control (Supplemental Figure 6A) or returned to normoxia for an additional 21 days prior to analysis (Figure 3, A and F). Tamoxifen treatment on hypoxia days 17–21 had no immediate effect on distal muscularization, RVSP, or RV weight ratio (Supplemental Figure 6, B–D). Most importantly, however, despite re-normoxia, tamoxifen-induced mice retained distal arteriole muscularization, PH, and RVH (Figure 3, F–I), suggesting that downregulation of EC HIFα is requisite for re-normoxia–induced reversal of these pathological findings.

### EC Hif1a or Hif2a deletion attenuates distal muscularization and PH with continued hypoxia.

**Figure 4:**
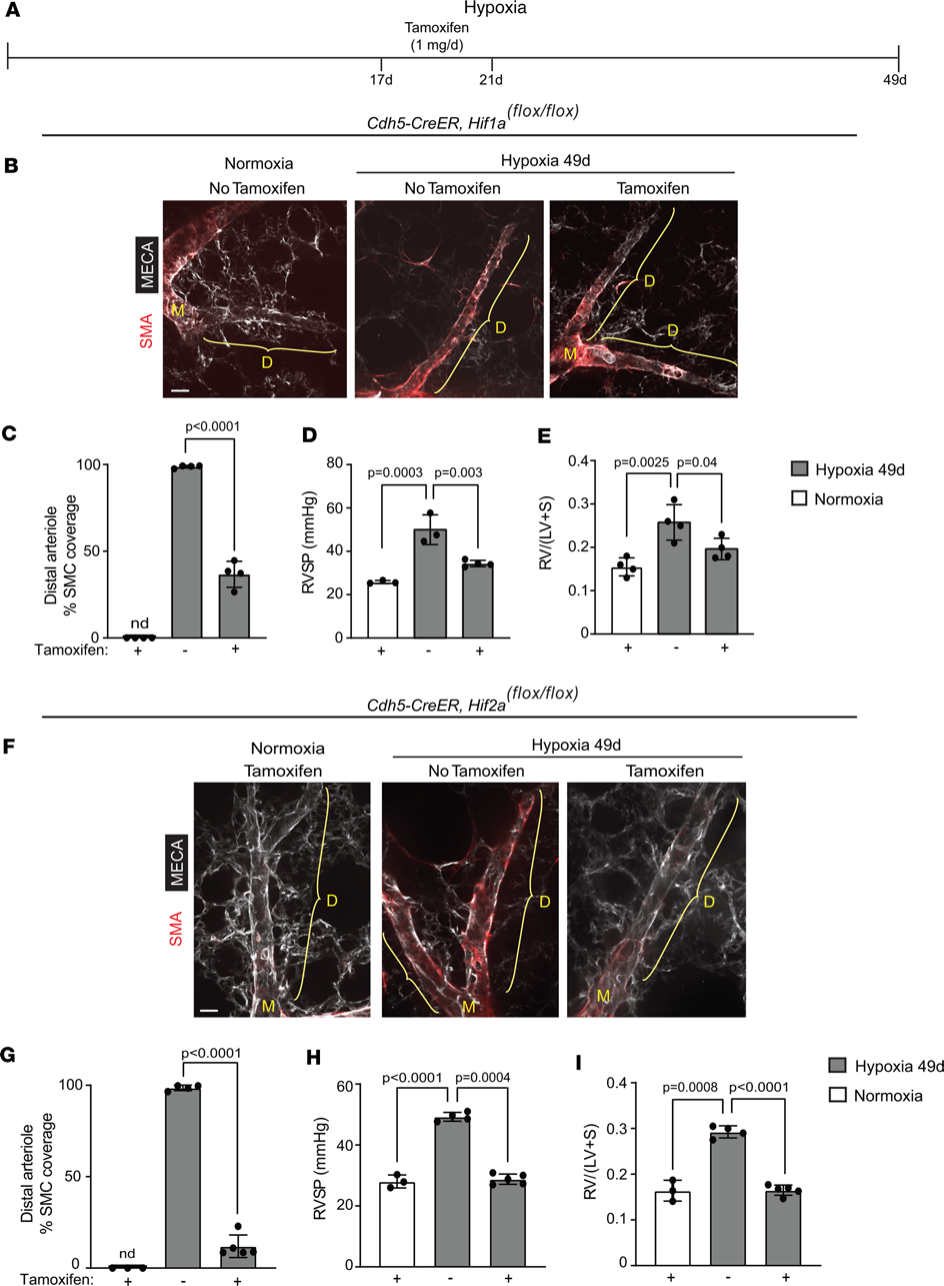
EC deletion of *Hif1a* or *Hif2a* attenuates established distal arteriole muscularization and PH. (**A**) Experimental strategy for **B**–**I**. (**B**–**I**) *Cdh5-CreER^T2^* mice carrying *Hif1a^fl/fl^* (**B**–**E**) or *Hif2a^fl/fl^* (**F**–**I**) were exposed to hypoxia for 49 days and tamoxifen (1 mg/day) was or was not administered on hypoxia days 17–21. (**B** and **F**) Vibratome lung sections were stained for SMA and MECA-32. (**C**–**E** and **G**–**I**) Percentage of SMC coverage of distal arterioles, RVSP, and RV weight ratio were measured as indicated. *n* = 3–5 mice (2 males, 1–3 females) per experimental group and 3 arterioles per mouse. Significance assessed by multifactor ANOVA with Tukey’s multiple-comparison test. nd, not detected. Scale bars: 20 μm.

To complement the EC HIFα gain-of-function experiments, we next pursued a *Hifa* isoform–specific loss-of-function approach. *Cdh5-CreER^T2^* mice carrying *Hif1a^fl/fl^* or *Hif2a^fl/fl^* were exposed to hypoxia (or normoxia as a control) for 49 days, and tamoxifen (1 mg/day) was or was not administered on hypoxia days 17–21 (Figure 4A). Tamoxifen-induced EC deletion of either *Hifa* isoform led to a reversal of distal pulmonary arteriole muscularization, RVSP, and RVH despite continued hypoxia (Figure 4, B–I). These results indicate that both HIFα isoforms are required in ECs for retention of hypoxia-induced pathological pulmonary vascular remodeling. As a control, *Cdh5-CreER^T2^* *Hif1a^fl/fl^* mice were exposed to normoxia, 17 days of hypoxia, or 21 days of hypoxia, with concomitant daily tamoxifen injections on days 17–21 (Supplemental Figure 6A). The 2 hypoxic groups showed equivalent distal arteriole muscularization, confirming that distal muscularization is essentially complete by day 17 and that *Hif1a* deletion on days 17–21 does not alter the phenotype on day 21 (Supplemental Figure 6E). Importantly, in *Cdh5-CreER^T2^* mice carrying *Hif1a^fl/fl^* or *Hif2a^fl/fl^* and exposed to hypoxia for 31 days, tamoxifen treatment on days 17–21 markedly reduced protein levels of the floxed-*Hifa* isoform in lung ECs (Supplemental Figure 7).

### EC-derived PDGF-B is required for sustaining distal arteriole muscularization.

**Figure 5:**
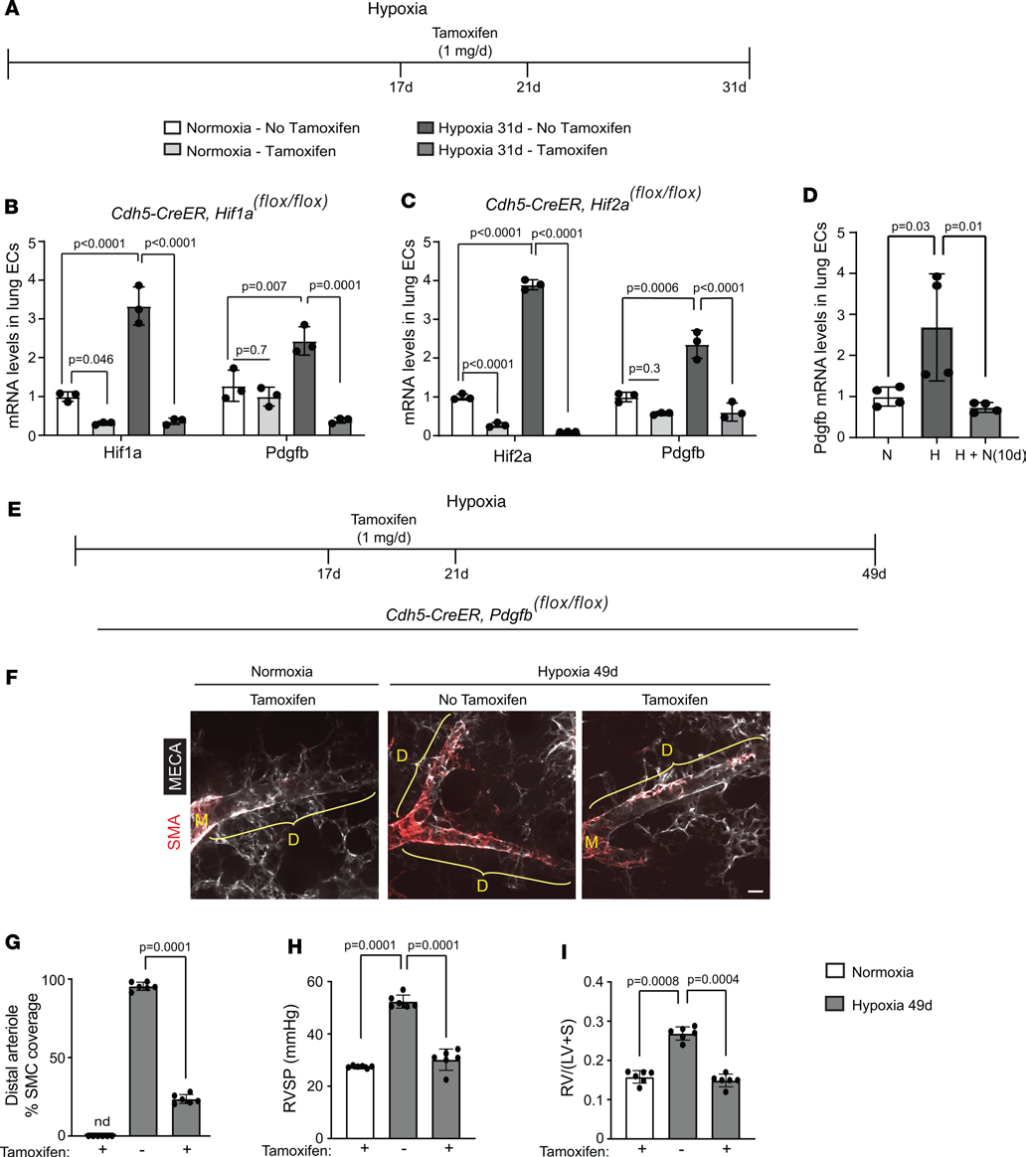
Deletion of EC *Pdgfb* attenuates established distal arteriole muscularization and PH. (**A**) Experimental strategy for **B** and **C**. (**B** and **C**) *Cdh5-CreER^T2^* mice carrying *Hif1a^fl/fl^* or *Hif2a^fl/fl^* were treated with hypoxia for 31 days and tamoxifen (1 mg/day) was or was not administered on hypoxia days 17–21. At 31 days, ECs were isolated by FACS, and *Hif1a*, *Hif2a*, and *Pdgfb* transcript levels were measured by qRT-PCR. *n* = 3 mice (2 males, 1 female) per experimental group. (**D**) Wild-type mice were exposed to normoxia (N) or hypoxia (H) for 21 days followed by normoxia for 10 days, and ECs were isolated by FACS. *Pdgfb* transcript levels were measured by qRT-PCR. *n* = 4 mice (2 males, 2 females) per experimental group. (**E**) Experimental strategy for **F**–**I**. (**F**) *Cdh5-CreER^T2^* *Pdgfb^fl/fl^* mice were exposed to hypoxia for 49 days, and tamoxifen (1 mg/day) was or was not administered on hypoxia days 17–21. Vibratome lung sections were stained for SMA and MECA-32. (**G**–**I**) Percentage of distal arterioles covered by SMCs, RVSP, and RV weight ratio were measured, respectively. *n* = 6 mice (3 males, 3 females) per experimental group and 3 arterioles per mouse. nd, not detected. Significance assessed by multifactor ANOVA with Tukey’s multiple-comparison test (**B**–**D** and **G**–**I**). Scale bar: 20 μm.

We previously found that hypoxia-induced EC expression of PDGF-B induces distal muscularization and PH and that EC deletion of *Hif1a* attenuates lung PDGF-B levels induced by subsequent hypoxia exposure (10). Tamoxifen treatment on hypoxia days 17–21 markedly reduced levels of the floxed-*Hifa* isoform in lung ECs isolated by FACS from *Cdh5-CreER^T2^* mice also carrying *Hif1a^fl/fl^* or *Hif2a^fl/fl^* on hypoxia day 31, and most importantly downregulated lung EC *Pdgfb* levels (Figure 5, A–C). Furthermore, whole-lung lysates or isolated lung ECs from wild-type mice exposed to hypoxia had upregulated *Pdgfb* that was reduced with re-exposure to normoxia (Figure 5D and Supplemental Figure 8, A and B). These data suggest that EC-derived PDGF-B may play a key role in sustaining pathological arteriole remodeling under chronic hypoxia. To evaluate this hypothesis, *Cdh5-CreER^T2^ Pdgfb^fl/fl^* mice were exposed to hypoxia for 31 or 49 days and tamoxifen (1 mg/day) was or was not administered on hypoxia days 17–21 (Figure 5E and Supplemental Figure 8C). This tamoxifen treatment induced a marked reduction of lung EC *Pdgfb* on hypoxia day 31 (Supplemental Figure 8D) and of distal muscularization, RVSP, and RV weight ratio on day 49 (Figure 5, F–I). Taken together, these findings indicate that an intact and activated EC HIFα/PDGF-B axis is required for retaining pathological distal muscularization and PH under chronic hypoxia.

### EC-derived PDGF-B induces SMC Beclin1.

**Figure 6:**
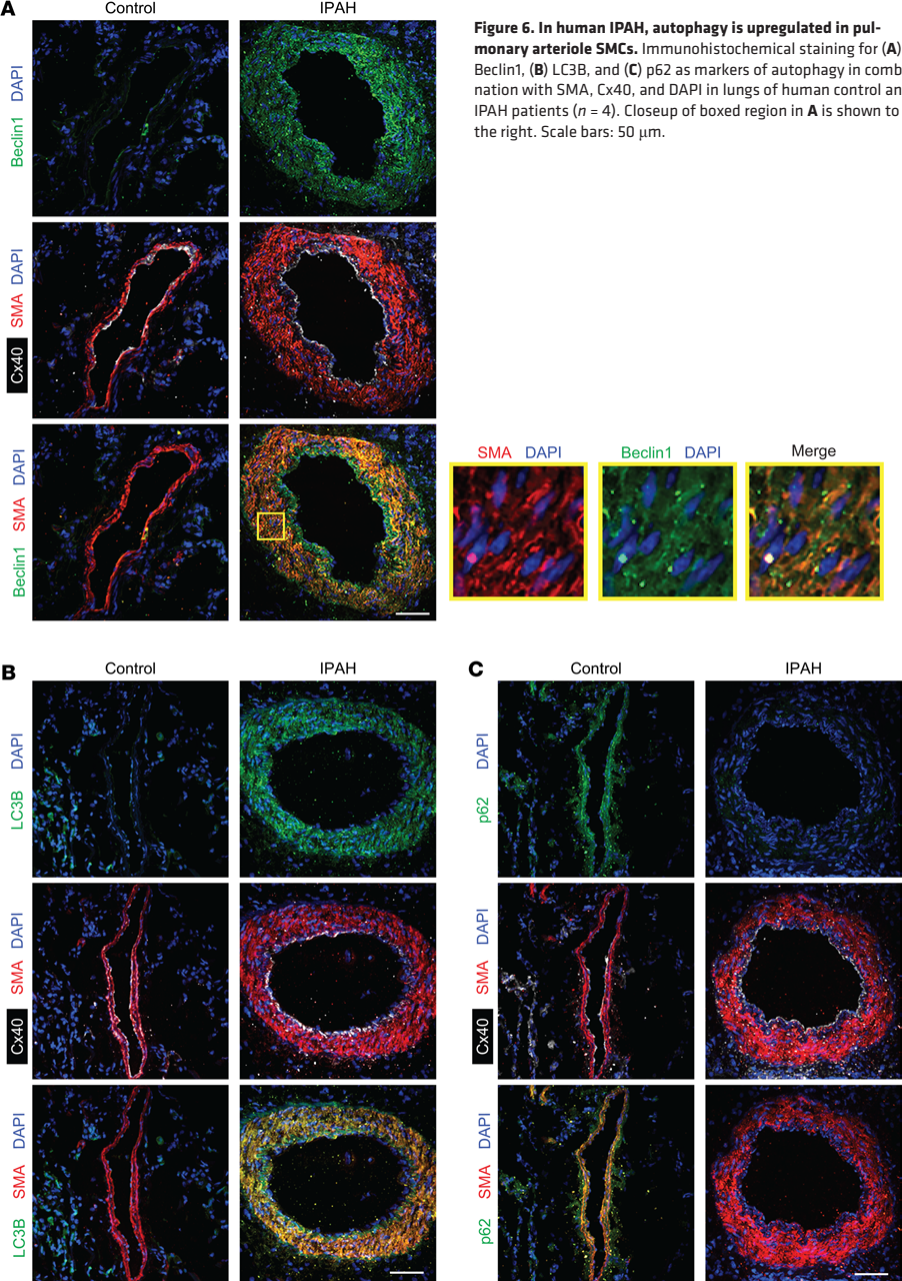
In human IPAH, autophagy is upregulated in pulmonary arteriole SMCs. Immunohistochemical staining for (**A**) Beclin1, (**B**) LC3B, and (**C**) p62 as markers of autophagy in combination with SMA, Cx40, and DAPI in lungs of human control and IPAH patients (*n* = 4). Closeup of boxed region in **A** is shown to the right. Scale bars: 50 μm.

**Figure 7:**
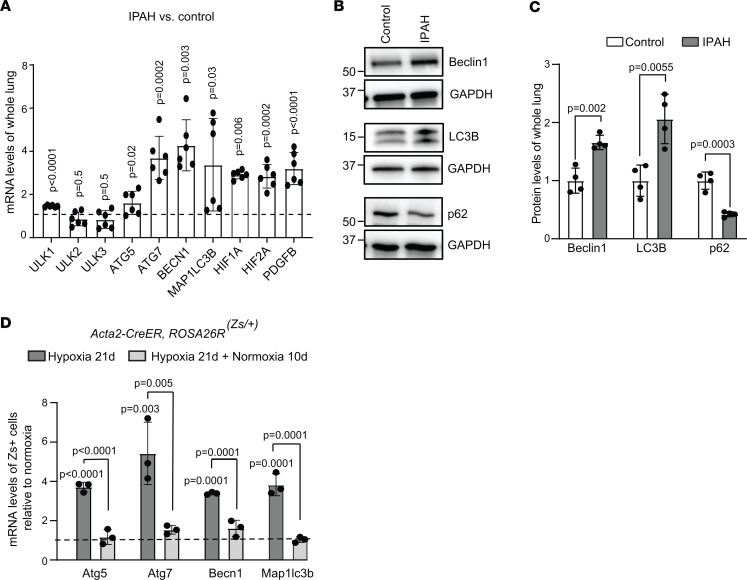
Autophagy-related genes are upregulated in lungs of IPAH humans and lung SMCs of hypoxic mice and downregulated in murine lung SMCs with re-normoxia. (**A**) qRT-PCR analysis of autophagy-related gene products and *HIF1A*, *HIF2A*, and *PDGFB* from lung lysates of patients with IPAH compared with that of control individuals (*n* = 6). (**B**) Western blots of lung lysates of IPAH patients and controls probed for Beclin1, LC3B, p62, and GAPDH. (**C**) Densitometry of protein bands shown in **B** relative to GAPDH and normalized to control (*n* = 4). (**D**) *Acta2-CreER^T2^* *ROSA26R^Zs/+^* mice were induced with tamoxifen, rested for 5 days, exposed to normoxia or hypoxia for 21 days or to hypoxia for 21 days, followed by re-normoxia for 10 days. Lung Zs^+^ cells were isolated by FACS, and the expression of autophagy genes *Atg5*, *Atg7*, *Becn1*, and *Map1lc3b* was analyzed by qRT-PCR and normalized to normoxia. *n* = 3 mice (1 male, 2 females) per experimental group. Significance assessed by 2-tailed Student’s *t* test (**A** and **C**) or multifactor ANOVA with Tukey’s multiple-comparison test (**D**).

PDGF-B treatment of SMCs induces autophagy and cell survival (21, 22). In the lungs of hypoxia- and/or monocrotaline-exposed rats, critical components of the autophagic pathway, ATG5, ATG7, Beclin1, and LC3B, are markedly increased and p62, which is downregulated during autophagy, is decreased (23, 24, 36). LC3B expression is increased in human PH lung tissues (37). Herein, immunohistochemical staining of lung tissue revealed a marked upregulation of LC3B and Beclin1 and downregulation of p62 in SMCs in PAs of idiopathic PAH (IPAH) patients compared with human controls (Figure 6 and Supplemental Table 1). Furthermore, mRNA and/or protein quantification (Supplemental Tables 2 and 3) of autophagy-related genes showed similar increases in *MAP1LC3B* (LC3B) and Beclin1 as well as ULK1, ATG5, and ATG7 and downregulation of p62 (Figure 7, A–C). HIFα isoforms and PDGF-B were also upregulated in IPAH lungs compared with controls. These data suggest an activation of autophagy in the human IPAH lung. Next, we examined autophagy during reversal of hypoxia-induced remodeling. *Acta2-CreER^T2^ ROSA26R^Zs/+^* mice were induced with tamoxifen, rested, exposed to hypoxia for 21 days, and then re-normoxia for 10 days. Lung Zs^+^ SMCs were isolated by FACS, and the expression of *Atg5*, *Atg7*, *Becn1*, and *Map1lc3b* was increased with hypoxia and decreased with re-normoxia (Figure 7D), suggesting a role for autophagy-related genes in the retention of pathological distal arteriole muscularization.

**Figure 8:**
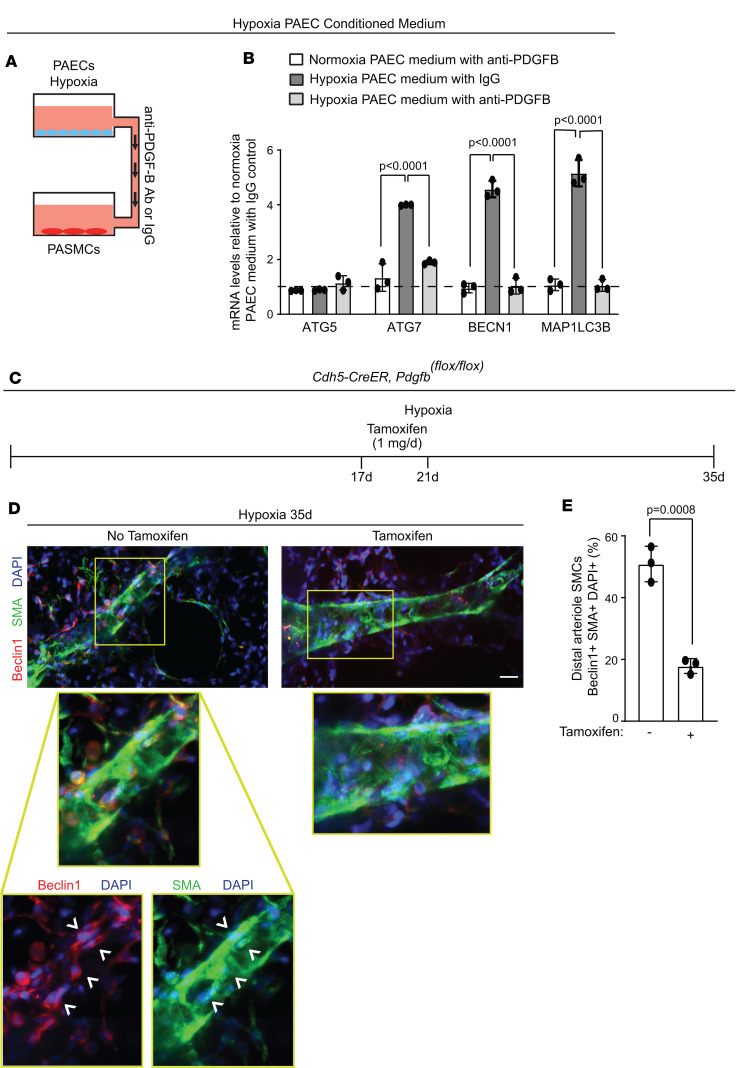
Pharmacological or genetic inhibition of EC-derived PDGF-B in mice or in culture, respectively, downregulates Beclin1 in SMCs. (**A**) Experimental strategy for **B**. (**B**) Human PAECs were exposed to normoxia or hypoxia (3% O_2_) for 16 hours, and the conditioned medium was collected and pretreated with either anti–PDGF-B blocking antibody or IgG isotype control for 1 hour. Human PASMCs were incubated with the pretreated PAEC-conditioned medium in normoxic conditions for 48 hours, and then qRT-PCR was used to assess mRNA levels of *ATG5*, *ATG7*, *BECN1*, and *MAP1LC3B* in the PASMCs. Transcript levels relative to *18S* rRNA were normalized to normoxia PAEC medium treated with IgG (dashed line). *n* = 3. Significance assessed by multifactor ANOVA with Tukey’s multiple-comparison test. (**C**) Experimental strategy for **D** and **E**. (**D**) *Cdh5-CreER^T2^* *Pdgfb^fl/fl^* mice were exposed to hypoxia for 35 days and tamoxifen (1 mg/day) was or was not administered on hypoxia days 17–21. Vibratome lung sections were stained for Beclin1, SMA, and nuclei (DAPI). Closeups of boxed region are shown below. Arrowheads indicate Beclin1^+^SMA^+^ cells. (**E**) Quantification of the percentage of distal arteriole SMCs that are Beclin1^+^. *n* = 3 mice (2 males, 1 female) per experimental group, 3–4 arterioles analyzed per mouse. Significance assessed by 2-tailed Student’s *t* test. Scale bar: 20 μm.

Given the non–cell-autonomous inductive effect of EC HIFα/PDGF-B on pathological SMC expansion in chronic hypoxia (10), we next assessed the effects of PDGF-B on autophagy-related genes. PDGF-B treatment of human PASMCs upregulated *ATG5*, *ATG7*, *BECN1*, and *MAP1LCB* transcripts levels (Supplemental Figure 9A), without altering the rate of *BECN1* mRNA decay (Supplemental Figure 9B), suggesting that *BECN1* induction by PDGF-B is not regulated by degradation, but instead by transcription. To assess the effects of hypoxia-induced EC-conditioned medium on autophagy-related genes in PASMCs, human PAECs were exposed to hypoxia (3% O_2_) for 6 or 16 hours and then the medium was collected and added to human PASMCs under normoxic conditions for 48 hours (Supplemental Figure 9C). At both time points, mRNA levels of EC *Pdgfb* and PASMC *ATG7*, *BECN1*, and *MAP1LC3B* were upregulated (Supplemental Figure 9, D and E). Furthermore, the hypoxic PAEC-conditioned medium was pretreated with anti–PDGF-B blocking antibody or IgG isotype control and then added to PASMCs under normoxic conditions for 48 hours (Figure 8A). Transcript levels of *ATG7*, *BECN1*, and *MAP1LC3B* in PASMCs were downregulated with anti–PDGF-B treatment compared with control (Figure 8B). Thus, these findings suggest that hypoxia-induced EC PDGF-B upregulates autophagy in SMCs.

Beclin1 expression is increased in PAs of mice exposed to hypoxia and is correlated with autophagy in experimental (23, 24) and human PH (see Figures 6 and 7). Herein, *Cdh5-CreER^T2^ Pdgfb^fl/fl^* mice were exposed to hypoxia for 35 days and tamoxifen (1 mg/day) was or was not administered on hypoxia days 17–21 (Figure 8C). Tamoxifen-induced *Pdgfb* deletion reduced the percentage of Beclin1^+^ distal arteriole SMCs from 51% ± 3% to 18% ± 1% (Figure 8, D and E).

**Figure 9:**
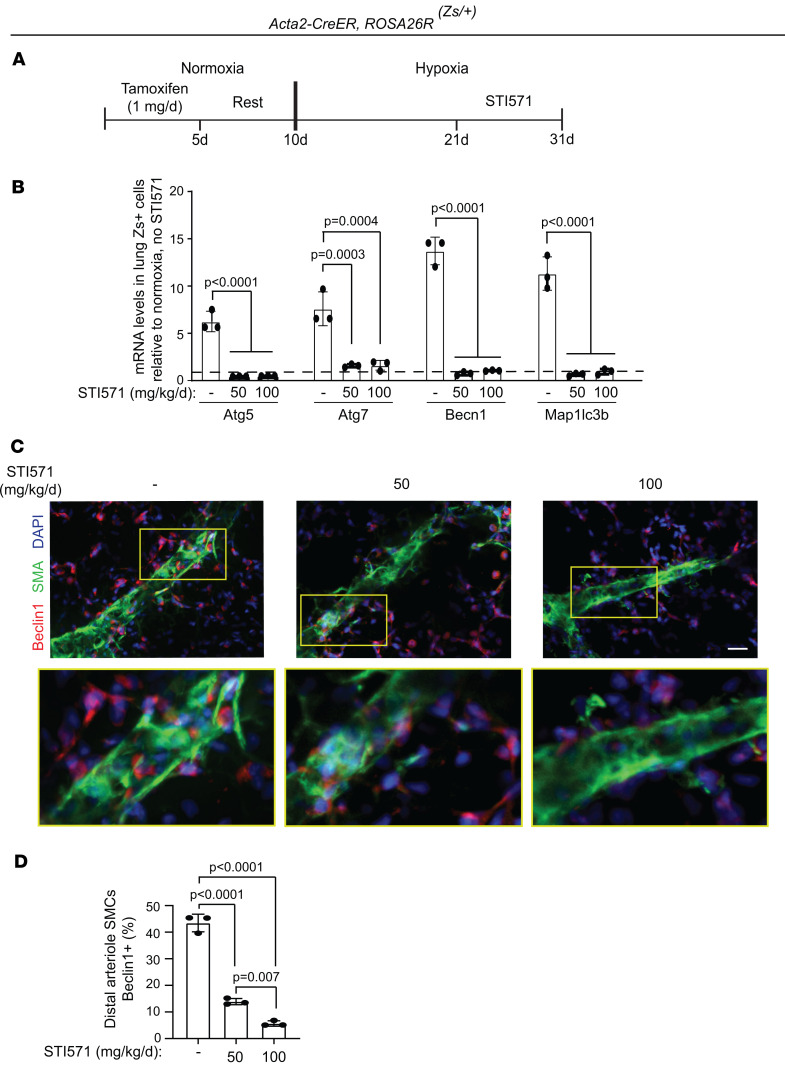
Inhibition of tyrosine receptor kinases, including PDGFRs, attenuates lung SMC Beclin1 levels. (**A**) Experimental strategy for **B**–**D**. (**B**) *Acta2-CreER^T2^* *ROSA26R^Zs/+^* mice were exposed to hypoxia or normoxia for 31 days and STI571 was administered at 0, 50, or 100 mg/kg/d by daily intraperitoneal injections on hypoxia days 21–31. Lung Zs^+^ SMCs were isolated by FACS, and expression levels of *Atg5*, *Atg7*, *Becn1*, and *Map1lc3b* with hypoxia relative to normoxia, no STI571 were analyzed by qRT-PCR. *n* = 3 mice (2 males, 1 female) per experimental group. (**C**) Vibratome lung sections were stained for Beclin1, SMA, and nuclei (DAPI). Closeups of boxed regions are shown below. (**D**) Quantification of the percentage of distal arteriole SMCs that are Belclin1^+^. *n* = 3 mice (1 male, 2 female) per experimental group, 3–4 arterioles analyzed per mouse. Significance assessed by multifactor ANOVA with Tukey’s multiple-comparison test (**B** and **D**). Scale bar: 20 μm.

As a therapeutic correlate, we assessed the effects of STI571, which targets the ATP binding site of tyrosine kinases, including PDGFRs, on SMC Beclin1 expression. We initially confirmed that STI571 treatment reverses established vascular remodeling in rodent experimental models (38) (Supplemental Figure 10). Next, *Acta2-CreER^T2^ ROSA26R^Zs/+^* mice were induced with tamoxifen and exposed to hypoxia for 31 days, and STI571 (0, 50, or 100 mg/kg/d) was administered by daily intraperitoneal injections from hypoxia day 21 to 31 (Figure 9A). mRNA levels of *Atg5*, *Atg7*, *Becn1*, and *Map1lc3b* in lung Zs^+^ SMCs isolated by FACS were significantly decreased in hypoxic mice treated with STI571 compared with no STI571 treatment (Figure 9B). In addition, immunohistochemical analysis indicated that STI571 treatment markedly reduced the percentage of distal arteriole SMCs that were Beclin1^+^ in a dose-dependent manner (Figure 9, C and D). Notably, in the more severe pulmonary vascular disease model of combined hypoxia and weekly Sugen 5416 administration (39), STI571 treatment altered mRNA levels of markers of autophagy (decreases) and apoptosis (increases) in lung Zs^+^ SMCs (Supplemental Figure 11, A–C) and reduced the percentage of Beclin1^+^ distal arteriole SMCs (Supplemental Figure 11, D–F). These results suggest that tyrosine kinase inhibition plays a role previously not described in reducing SMC autophagy to reverse established distal arteriole muscularization.

### SMC Beclin1 is required for the retention of distal arteriole muscularization and PH.

**Figure 10:**
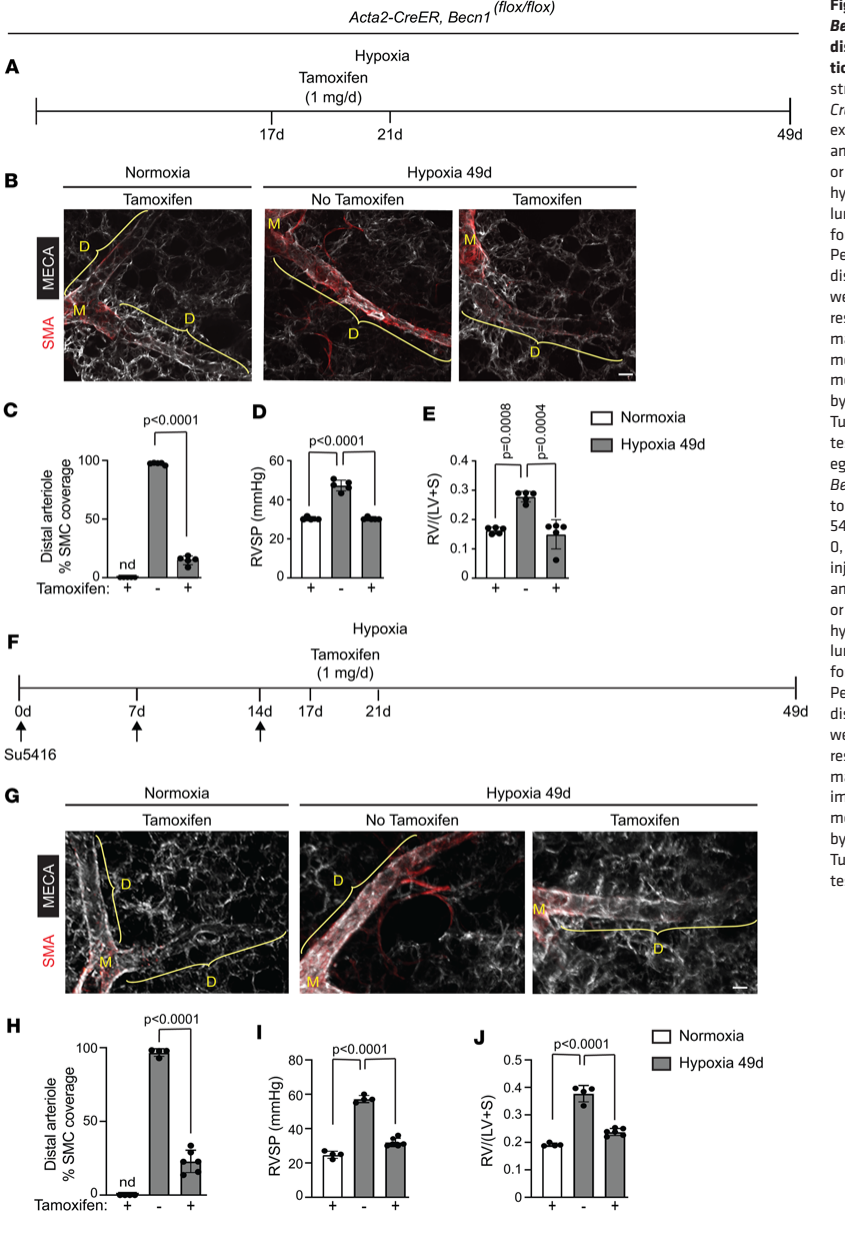
SMC deletion of *Becn1* attenuates established distal arteriole muscularization and PH. (**A**) Experimental strategy for **B**–**E**. (**B**) *Acta2-CreER^T2^* *Becn1^fl/fl^* mice were exposed to hypoxia for 49 days and tamoxifen (1 mg/day) was or was not administered on hypoxia days 17–21. Vibratome lung sections were stained for SMA and MECA-32. (**C**–**E**) Percentage of SMC coverage of distal arterioles, RVSP, and RV weight ratio were measured, respectively. *n* = 5 mice (3 males, 2 females) per experimental group, 3 arterioles per mouse. Significance assessed by multifactor ANOVA with Tukey’s multiple-comparison test. (**F**) Experimental strategy for **G**–**J**. (**G**) *Acta2-CreER^T2^* *Becn1^fl/fl^* mice were exposed to hypoxia for 49 days. Sugen 5416 was administered on days 0, 7, and 14 by subcutaneous injection (20 mg/kg/dose), and tamoxifen (1 mg/day) was or was not administered on hypoxia days 17–21. Vibratome lung sections were stained for SMA and MECA-32. (**H**–**J**) Percentage of SMC coverage of distal arterioles, RVSP, and RV weight ratio were measured, respectively. *n* = 4–6 mice (2 males, 2–4 females) per experimental group, 3 arterioles per mouse. Significance assessed by multifactor ANOVA with Tukey’s multiple-comparison test. Scale bars: 20 μm.

Thus far, we have shown that the EC HIFα/PDGF-B axis is important for sustaining established distal arteriole muscularization under continuous hypoxia and that EC-derived PDGF-B induces expression of SMC Beclin1 and other autophagy genes. To evaluate the specific role of SMC Beclin1 in sustaining pulmonary vascular remodeling and PH, *Acta2-CreER^T2^ Becn1^fl/fl^* mice were treated with hypoxia for 35 or 49 days, and tamoxifen was or was not administered on hypoxia days 17–21 (Figure 10A and Supplemental Figure 12A). This tamoxifen treatment induced a marked reduction in the percentage of distal arteriole SMCs that were Beclin1^+^ on day 35 (Supplemental Figure 12, B and C) and by day 49, reversed hypoxia-induced distal muscularization, PH, and RVH (Figure 10, B–E). Additionally, mice of the same genotype were exposed to hypoxia for 49 days with injections of Sugen 5416 on day 0, 7, and 14, and tamoxifen was or was not administered on hypoxia days 17–21 (Figure 10F). Similar to the hypoxia model (Figure 10, A–E), with Sugen 5416/hypoxia treatment, deletion of *Becn1* in SMCs reversed established distal arteriole muscularization, PH, and RVH (Figure 10, G–J). In contrast, *Becn1* deletion in ECs did not alter pulmonary vascular remodeling in mice exposed to chronic hypoxia (Supplemental Figure 13). Thus, Beclin1 in SMCs, but not ECs, is requisite for sustaining pathological distal muscularization and PH.

**Figure 11:**
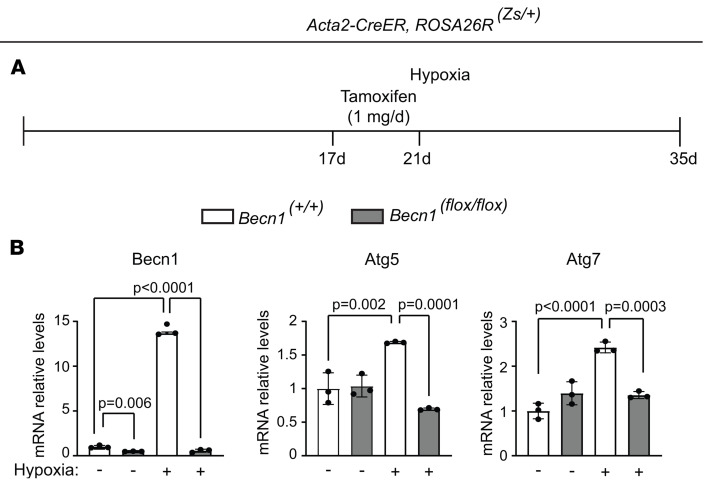
*Becn1* deletion in SMCs attenuates hypoxia-induced increase in *Atg5* and *Atg7*. (**A**) Experimental strategy for **B**. (**B**) *Acta2-CreER^T2^* *ROSA26R^Zs/+^* mice also carrying *Becn1^fl/fl^* or *Becn1^+/+^* were exposed to hypoxia for 35 days, and tamoxifen (1 mg/day) was administered on days 17–21. Zs^+^ cells were isolated by FACS, and mRNA levels of *Becn1*, *Atg5*, and *Atg7* were measured by qRT-PCR and normalized to *Becn1^+/+^*, normoxia. *n* = 3 mice (2 males, 1 female) per experimental group. Significance assessed by multifactor ANOVA with Tukey’s multiple-comparison test. nd, not detected.

Cross-talk between autophagy and apoptosis is regulated by Beclin1. For instance, as the binding partner for the antiapoptotic mediator Bcl2, Beclin1 is critical as to whether cells are resistant to apoptosis or autophagy (40). Beclin1-deficient mice display embryonic lethality and neuronal apoptosis, suggesting that Beclin1 is associated with cell survival (41, 42). Herein, *Acta2-CreER^T2^ Becn1^fl/fl^* mice were treated with hypoxia for 35 days, and tamoxifen was or was not administered on hypoxia days 17–21 (Supplemental Figure 14A). *Becn1* deletion in SMCs reduced the percentage of distal arteriole SMCs that were ATG7^+^ by more than 50% (Supplemental Figure 14, B and C). We next used *Acta2-CreER^T2^ ROSA26R^Zs/+^* mice carrying *Becn1^fl/fl^* or *Becn1^+/+^* to evaluate the effect of *Becn1* deletion in SMCs on hypoxia-induced *Atg5* and *Atg7* expression. These mice were treated with hypoxia for 35 days, and tamoxifen was administered on hypoxia days 17–21 (Figure 11A). *Becn1* deletion attenuated the hypoxia-induced increase in *Atg5* and *Atg7* transcript levels (Figure 11B).

**Figure 12:**
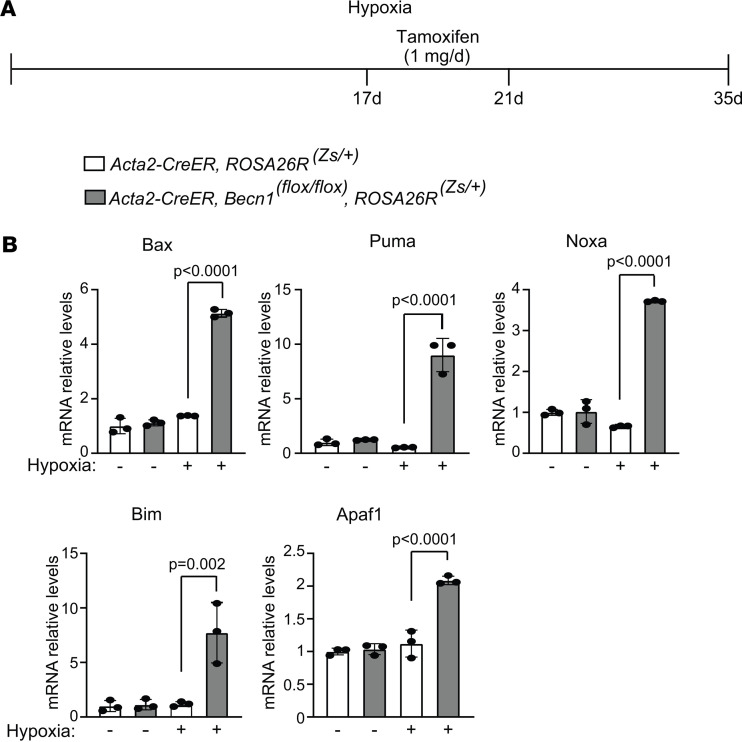
Proapoptosis markers are upregulated by SMC deletion of *Becn1*. (**A**) Experimental strategy for **B**. (**B**) *Acta2-CreER^T2^* *ROSA26R^Zs/+^* mice carrying *Becn1^fl/fl^* or *Becn1^+/+^* were exposed to hypoxia for 35 days and tamoxifen (1 mg/day) was administered on hypoxia days 17–21. Zs^+^ cells were isolated by FACS, and the mRNA levels of proapoptosis markers were measured by qRT-PCR. *n* = 3 mice (2 males, 1 female) per experimental group. Significance assessed by multifactor ANOVA with Tukey’s multiple-comparison test.

**Figure 13:**
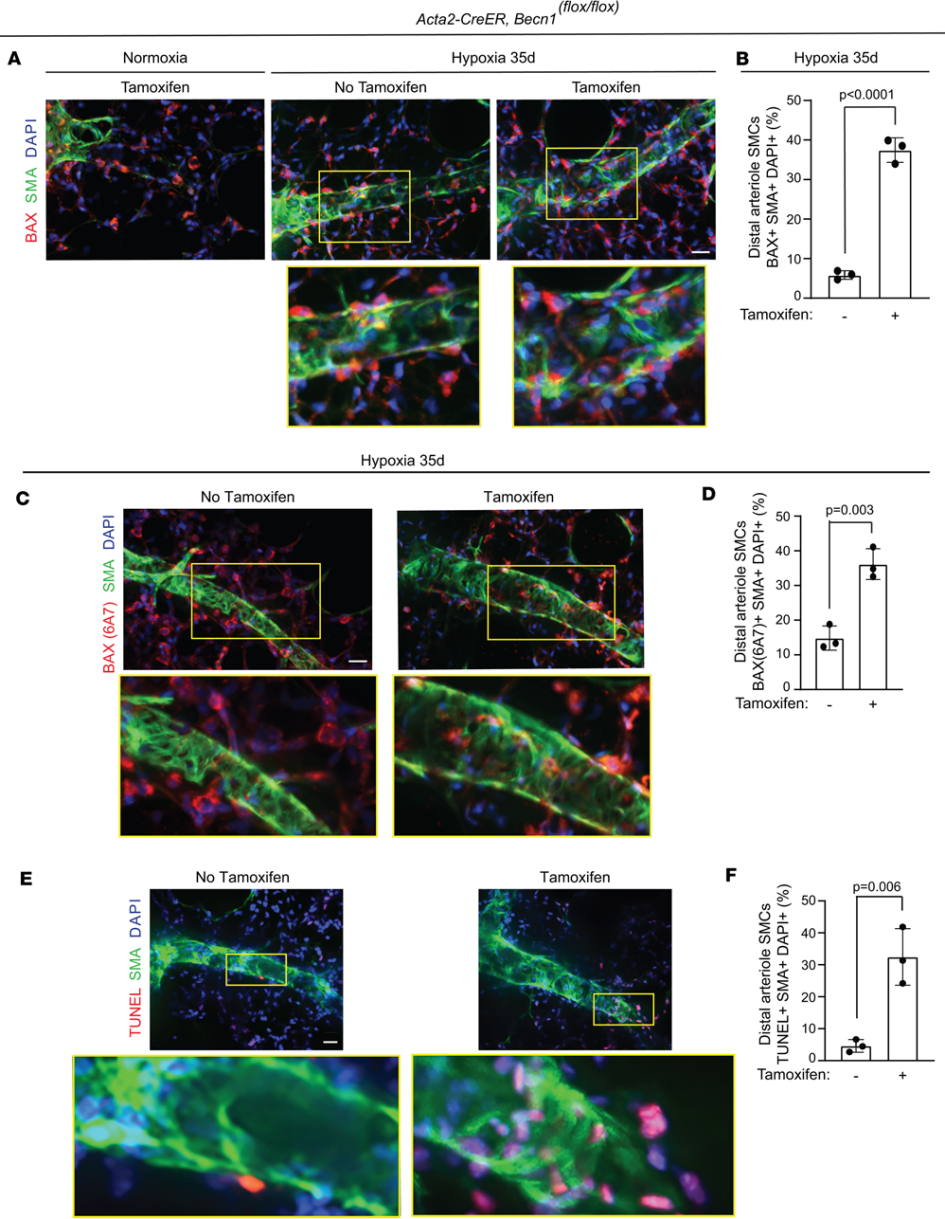
Deletion of *Becn1* in SMCs induces apoptosis of distal arteriole SMCs. (**A**–**F**) *Acta2-CreER^T2^* *Becn1^fl/fl^* mice were exposed to hypoxia for 35 days (or to normoxia in **A**, left panel) and tamoxifen (1 mg/day) was or was not administered on hypoxia days 17–21. Vibratome lung sections were stained for SMA, nuclei (DAPI), and BAX (**A**), activated BAX (6A7) (**C**), or TUNEL (**E**). Closeups of boxed regions are shown below. The percentage of distal arteriole SMCs expressing BAX (**B**), activated BAX (**D**), or TUNEL (**F**) was quantified. *n* = 3 mice (2 males, 1 female) per experimental group, 3–4 arterioles analyzed per mouse. Significance assessed by 2-tailed Student’s *t* test. Scale bars: 20 μm.

Finally, the effect of *Becn1* deletion in SMCs on apoptosis and cell proliferation was analyzed. *Acta2-CreER^T2^ ROSA26R^Zs/+^* mice carrying *Becn1^fl/fl^* or *Becn1^+/+^* were treated with hypoxia for 35 days, and tamoxifen was administered on hypoxia days 17–21 (Figure 12A and Supplemental Figure 15A). Lung Zs^+^ SMCs were isolated by FACS and analyzed by qRT-PCR for transcripts of proapoptotic genes *Bax*, *Puma*, *Noxa*, *Bim*, and *Apaf1*, the proproliferative gene *Ccna*, and antiproliferative genes *p21* and *p27*. Tamoxifen-induced *Becn1* deletion led to a significant increase in levels of the proapoptotic and antiproliferative mRNAs and reduction in *Ccna* mRNA with hypoxia (Figure 12B and Supplemental Figure 15B). Additionally, *Acta2-CreER^T2^ Becn1^fl/fl^* mice were treated with hypoxia for 35 days, and tamoxifen was or was not administered on hypoxia days 17–21. Immunohistochemistry indicated that *Becn1* deletion resulted in an approximately 6.5-fold or approximately 2.5-fold increase in the percentage of distal arteriole SMCs that stained with antibodies against total BAX or activated (6A7) BAX (Figure 13, A–D), respectively, suggesting that *Becn1* deficiency in SMCs induces cell death to reverse established distal arteriole muscularization. As a confirmation, tamoxifen treatment of *Acta2-CreER^T2^ Becn1^fl/fl^* mice increased the percentage of TUNEL^+^ distal arteriole SMCs by approximately 8-fold (Figure 13, E and F). Additionally, immunohistochemistry indicated that *Becn1* deletion decreased the percentage of distal pulmonary arteriole SMCs that express Ki67 (Supplemental Figure 15, C and D). Taken together, these data suggest that *Becn1* deletion in SMCs reverses hypoxia-induced distal arteriole muscularization by promoting SMC apoptotic cell death and decreasing proliferation.

## Discussion

Excess SMCs are a critical component of many cardiovascular diseases, including SMC coating of normally unmuscularized distal pulmonary arterioles in PH. Mechanisms governing how pathological hypermuscularization is sustained are poorly understood. In this study, we leverage the reversal of hypoxia-induced distal muscularization that occurs with re-normoxia to delineate how pathological distal arteriole muscularization is retained in hypoxia-induced PH and how it can be attenuated. With normoxia after hypoxia exposure, our findings demonstrate sequential regression of distal pulmonary arteriole muscularization, PH, and RVH in mice, starting on day 14 of re-normoxia (Figure 1). The distal arteriole SMCs do not change fates with re-normoxia, but rather undergo apoptosis, which is in agreement with prior studies of re-normoxia in rats showing apoptosis of SMCs surrounding small arterioles (7, 8). Interestingly, we found that hypoxia induces distal muscularization despite a prior hypoxia and re-normoxia cycle, suggesting that a re-normoxia phase does not inhibit mechanisms underlying pathological muscularization. Another key finding is that the SMC loss with re-normoxia is limited to the distal arteriole region, suggesting that distal arteriole SMCs, which primarily derive from PDGFR-β^+^SMA^+^SMMHC^+^ progenitors (28), differ from SMCs in the more proximal regions of the arteriole in terms of sensitivity to re-normoxia. The nature of this sensitivity is not known, but warrants further analysis as it potentially has profound clinical ramifications.

Targeting HIF1-α or HIF2-α pharmacologically or genetically reduces chronic hypoxia-induced vascular remodeling (10, 12–15, 43–47). Although there are some discrepancies, during the adult onset of hypoxia-induced pulmonary vascular remodeling and PH, a pathological role of HIF2-α in ECs is generally more established than that of HIF1-α, whereas in SMCs, HIF1-α may contribute. Tamoxifen induction of *Hif1a^fl/fl^* mice also carrying *Pdgrb-CreER^T2^* (marking pericytes, fibroblasts and SMC progenitors in the lung; ref. 48) or *Myh11-CreER^T2^* attenuates hypoxia-induced pulmonary vascular disease (10, 14). In contrast, constitutive *Hif1a* deletion in SM22α^+^ cells increases PA pressure under normoxia and hypoxia (46). Our findings indicate that SMC HIF1-α is neither necessary nor sufficient to retain established distal pulmonary arteriole muscularization in hypoxia-induced PH (Figure 3 and Supplemental Figure 3). EC deletion of *Hif2a*, but not *Hif1a*, with *L1-Cre* or *Tie2-CreER^T2^* attenuates hypoxia-induced pulmonary vascular disease (15, 47). In contrast, *Cdh5-CreER^T2^* *Hif1a^fl/fl^* mice are protected against hypoxia-induced distal pulmonary arteriole muscularization and PH (10).

Compared with the roles of vascular cell–specific HIFα isoforms in induction of pulmonary vascular disease, their roles in sustaining pathological SMCs in PH is understudied and poorly understood. In the current study, although we did not directly compare the relative effects of EC HIF1-α and HIF2-α in retention and reversibility of established pulmonary vascular disease, our results suggest that with *Cdh5-CreER^T2^*, deletion of *Hif2a* reduces distal muscularization (by ~87%) more so than *Hif1a* deletion does (by ~62%) despite continued hypoxia (Figure 4). Furthermore, EC deletion of *Hif2a* restores PA pressure and the RV weight ratio to normoxic levels, whereas *Hif1a* EC deletion significantly reduces these hemodynamic parameters, but not to normoxic levels. As a complementary approach, studies with *Cdh5-CreER^T2^ Vhl^fl/fl^* mice, which have elevated EC HIFα levels under normoxia (10), indicate that EC HIFα induces the retention of pathological muscularization. Indeed, when these mice are injected with tamoxifen at the tail end of a 21-day hypoxia exposure and then returned to normoxia, distal arteriole muscularization, PH, and RVH persist (Figure 3). These results highlight that sustaining pathological distal muscularization is not a passive cell-intrinsic phenomenon of SMCs, but instead requires input from ECs.

Signaling by PDGF-B through PDGFR-β is central to PH pathogenesis. In the lung, PDGF-B is primarily synthesized by and released from ECs and circulating inflammatory cells, and increased levels of PDGF-B are reported in the blood and lung tissue of patients with PH (29, 49–52). In mice, lung EC *Pdgfb* levels are elevated by day 7 of hypoxia (10) and remain upregulated on day 31 compared with normoxia (Figure 5). Analysis of CD64^+^Ly6G^–^ macrophages in bronchoalveolar lavage fluid as well as in the residual lung (i.e., after removal of this fluid) from mice exposed to hypoxia shows that *Pdgfb* increases within 1 day of hypoxia and, in the case of the residual lung, peaks at day 3 (29). Hypoxia-induced distal muscularization is completely prevented in *Pdgfb^+/–^* mice and markedly attenuated by deletion of *Pdgfb* specifically in ECs or monocytes/macrophages (10, 28, 29). Herein, we demonstrate that deletion of EC *Pdgfb* in chronic hypoxia reverses established distal arteriole muscularization and PH (Figure 5). In contrast, reduction of lung macrophage *Pdgfb* levels does not reverse established distal arteriole muscularization (29). Taken together, these findings suggest that macrophage-derived PDGF-B may be primarily involved in the initial induction of hypoxia-mediated distal muscularization, whereas EC PDGF-B is critical in both the initiation and maintenance of distal muscularization.

It is interesting to compare these results to the role of the PDGF pathway in sustaining SMC-derived cells in atherosclerosis. Indeed, in *Apoe^–/–^* mice with established Western diet–induced atherosclerosis, *Pdgfrb* deletion with *Myh11-CreER^T2^* reduces fibrous cap SMA^+^ cells that are marked by the *Myh11-CreER^T2^* lineage by 50%, without altering the total number of SMA^+^ cells in the fibrous cap (53). Taken together, the findings suggest that when *Pdgfrb* is deleted in differentiated SMCs, a distinct population of cells contributes to retention of the atherosclerotic fibrous cap, whereas with *Pdgfb* deletion in ECs during PH of chronic hypoxia, pathological SMCs are lost and not replaced.

PDGF-B treatment of cultured SMCs induces autophagy, as assessed by autophagosome formation and expression of ATG5, ATG7, Beclin1, and LC3B, promoting cell survival (21, 22). Autophagy is a catabolic process that is essential to cellular homeostasis under stress, as it facilitates recycling of superfluous or dysfunctional organelles via lysosomal degradation (54). In human IPAH, LC3B is upregulated in lung lysates and large and small vessels of the lung (37). Our data from PAs of IPAH patients indicate that Beclin1 and LC3B are increased in EC and SMC layers and p62 is downregulated in SMCs (Figures 6 and 7). Furthermore, in addition to *BECN1* and *MAP1LC3B*, we found that transcript levels of *ULK1*, *ATG5*, *ATG7*, as well as *HIFA* and *PDGFB* are also upregulated in IPAH lung lysates. In diverse experimental rodent PH models, other groups have demonstrated that autophagy is enhanced in lung ECs, SMCs, and PAs (23, 24, 36, 37, 55). Herein, lung SMC levels of autophagy-related gene products, including *Becn1*, are demonstrated to be increased with hypoxia and downregulated with re-normoxia, suggesting that autophagy may be integral to retention of hypoxia-induced distal muscularization. Furthermore, data from culturing SMCs with conditioned medium from hypoxic ECs and in vivo studies of EC-specific deletion of *Pdgfb* or STI571 treatment suggest that under hypoxia with or without concomitant Sugen 5416, EC-derived PDGF-B induces autophagy-related transcripts in SMCs and specifically Beclin1 protein in distal pulmonary arteriole SMCs via a tyrosine kinase (most likely PDGFR-β) (Figures 8 and 9, and Supplemental Figure 11). These data suggest that hypoxia-mediated EC PDGF-B non–cell autonomously induces Beclin1 in pathological distal SMCs and perhaps, thereby promotes cell survival.

Beclin1 is a key member of a complex that is integral to major events during the autophagic process, ranging from formation of the autophagasome to autophagasome/endosome maturation (56). Beclin1 deficiency leads to defective autophagy, and *Becn1*-null embryos die by E7.5, with widespread cell death (25, 42). Our results indicate that SMC-specific deletion of *Becn1* reduces expression of ATG7 in distal pulmonary arteriole SMCs during chronic hypoxia and reverses distal muscularization and PH established by exposure to hypoxia with or without Sugen 5416, whereas no effect was observed with *Becn1* deletion in ECs (Figures 10 and 11, and Supplemental Figures 13 and 14). Notably, the lysosomal inhibitor chloroquine attenuates autophagy and degradation of bone morphogenetic protein type II receptor, a key player in human PAH, and has been shown to reverse pulmonary vascular remodeling and hemodynamics in monocrotaline- or hypoxia-induced PH in rats (23, 36). An important study in the field assessed the role of autophagy in inducing PH using mice with deletions of autophagy genes (37). Mice with global deletion of the *Map1lc3b* gene were shown to have exacerbated hypoxia-induced pulmonary arteriole wall thickening, PH, and RVH (37). This protective effect may be specific to LC3B, as *Becn1^+/–^* mice do not share a similar protection in response to hypoxia (37). In contrast, in a myocardial ischemia-reperfusion model, autophagy is elevated, and *Becn1^+/–^* mice have reduced autophagosome formation and myocardial injury (57). Thus, depending on the cardiovascular disease model, pathobiological effects of specific autophagic proteins differ.

The complex and intimate relationship between autophagy and apoptosis is critical to the pathophysiology of PH (36, 56, 58). Beclin1 plays an important role in cell survival and death, and although a number of pathways have been suggested to underlie its antiapoptotic effects in diverse tissues, a common mechanism is not established. Beclin1 is a regulator of the Bcl2 family of proteins, which includes key proapoptotic (e.g., Bax, Puma, Noxa, Bim) and antiapoptotic (e.g., Bcl-2) mediators (40, 56, 59), and is required for ATG5/ATG7–dependent autophagy. Knockdown of *Atg5* in PASMCs inhibits autophagy and proliferation and induces apoptosis (36, 56). Interestingly, deletion of *Becn1* in cortical and hippocampal neurons leads to enhanced activated caspase 3, suggestive of apoptosis, and severe neurodegeneration, concomitant with perturbations in both late endosome formation and phospholipid localization (41). In *C*. *elegans*, BEC-1, the ortholog of mammalian Beclin1, forms a complex with the antiapoptotic protein CED-9/Bcl-2, and BEC-1 depletion triggers caspase-dependent programmed cell death (60). Additionally, mice with *Becn1* deletion in adipocytes develop lipodystrophy and have elevated endoplasmic reticulum stress gene expression stimulating adipocyte apoptosis (61). Herein, following the establishment of hypoxia-induced pulmonary vascular remodeling, tamoxifen injection of *Acta2-CreER^T2^ Becn1^fl/fl^* mice reduces the level of autophagy markers and induces proapoptotic genes in lung SMCs (Figures 11 and 12). In distal arteriole SMCs, this treatment decreases ATG7 expression and increases levels of total and activated BAX and apoptosis (Figure 13 and Supplemental Figure 14), indicating that Beclin1 in SMCs prevents programmed cell death of established distal SMCs. Taken together, our studies support a model in which chronic hypoxia upregulates EC HIFα and thus, PDGF-B. In turn, PDGF-B induces SMC Beclin1 expression to promote the retention of established pulmonary distal arteriole SMCs, PH, and RVH, whereas re-normoxia reverses these processes. These findings pave the way for novel therapeutic strategies aimed at reversing established hypermuscularization and PH.

## Methods

### Sex as a biological variable.

As indicated in the figure legends, male and female mice were used in the studies.

### Animals and tamoxifen treatment.

Wild-type mice were of a C57BL/6 background. The mouse strains used were *Acta2-CreER^T2^* (62), *Cdh5-CreER^T2^* (63), *Bmx-CreER^T2^* (31), *ROSA26R^mTmG/mTmG^* (64), *ROSA26R^ZsGreen1/ZsGreen1^* (65), *Pdgfb^fl/fl^* (66), *Vhl^fl/fl^* (67), *Hif1a^fl/fl^* (68), *Hif2a^fl/fl^* (69), and *Becn1^fl/fl^* (41). Male and female mice aged 2–4 months and sex- and age-matched controls were used. Mice were exposed to hypoxia (FiO_2_ 10%) or normoxia for up to 49 days and then re-exposed to normoxia for up to 42 days. In select experiments, mice were again re-exposed to 21 days of hypoxia. For CreER-catalyzed recombination, mice were injected intraperitoneally with tamoxifen at 1 mg/day for 5 days, rested for 5 days, and then exposed to normoxia or hypoxia. In select experiments, mice were injected with tamoxifen (1 mg/day) on hypoxia days 17–21. For studies of more severe pulmonary vascular disease, during hypoxia exposure, mice were subcutaneously injected with Sugen 5416 (20 mg/kg/dose; MilliporeSigma, S8442) on hypoxia days 0, 7, and 14.

### Hypoxia treatment and hemodynamic measurements.

Mice were exposed to hypoxia (FiO_2_ 10%) in a rodent hypoxia chamber equipped with a calibrated oxygen controller and sensor (BioSpherix). RVSP was measured by inserting a catheter (Millar Instruments) into the RV via the right jugular vein. Subsequently, mice were sacrificed, and the heart was dissected with the weight ratio of the RV to the sum of the LV and septum assessed as previously described (27).

### STI571 administration.

*Acta2-CreER^T2^ ROSA26R^Zs/+^* mice were injected with tamoxifen, rested, and exposed to normoxia or hypoxia for 31 days. Starting on hypoxia day 21, mice received daily intraperitoneal injections of STI571 (imatinib mesylate, Selleckchem, S1026) at a dose of 50–100 mg/kg suspended in 0.9% sterile saline for 10 days, as previously described (38).

### Lung preparation for immunohistochemistry.

Lungs were prepared for immunohistochemistry as described previously (27). Briefly, mice were euthanized by isoflurane inhalation, and PBS was infused into the RV until the lungs turned a white color to flush the pulmonary vasculature. Lungs were then inflated with 2% low-melt agarose and incubated in ice-cold PBS for 30 minutes. Solidified agarose-filled lobes were immersed in Dent’s fixative (4:1, methanol/dimethyl sulfoxide) at 4°C overnight and then washed and stored in 100% methanol at –20°C. Lungs were bleached by incubating with 5% H_2_O_2_ in methanol for 10 minutes, sequentially rehydrated into PBS, and sectioned by vibratome at a thickness of 150 μm.

### Immunohistochemistry.

Vibratome lung sections were incubated with blocking buffer (5% normal goat serum in 0.5% Triton X-100/PBS [PBS-T]) at 4°C overnight. Sections were exposed to primary antibodies in blocking buffer for 1–3 days at 4°C, washed 3 times in PBS-T, and incubated in secondary antibodies overnight at 4°C. Sections were then washed 6 times in PBS-T and placed on slides in mounting media (Dako, S3023). Primary antibodies used were rat anti–MECA-32 (1:15; Developmental Studies Hybridoma Bank, AB-531797), rabbit anti-GFP (1:100; Invitrogen, A11122), rabbit anti-Beclin1 (1:100; Abcam, ab217179), rabbit anti-ATG7 (1:100; Abcam, ab133528), rabbit anti-BAX (1:100; Abcam, ab32503), anti–BAX (6A7) Alexa Fluor 546 (1:100; Santa Cruz, sc-23959), and/or anti-SMA directly conjugated to Cy3 or FITC (1:250; Sigma-Aldrich, C6198 or F3777, respectively). Secondary antibodies were conjugated to Alexa Fluor 488, Alexa Fluor 546, and Alexa Fluor 647 (1:250; Invitrogen, A11008, A11010, and A21247, respectively). Nuclei were stained with DAPI (1:500; Sigma-Aldrich, D9542). TUNEL assay was performed as per the “In Situ Cell Death Detection, TMR red” protocol (Roche Diagnostics, 12156792910).

As in prior studies (10, 27–29), immunohistochemical analysis focused on 3 vascular beds located in the cranial and medial aspects of the adult left lung and categorized arteriole segments in each of these vascular beds (by both position in the vascular tree and lumen diameter) as proximal (>75 μm diameter), middle (25–75 μm diameter), or distal (<25 μm diameter). The arteriole beds studied are in proximity to airway branches L.L1.A1.M1, L.L1.A1.L1, and L.L2.M2 (L, left main bronchus; L1, L2, L3 lateral branches; M1, M2, medial branches; A1, A2, anterior branch; Supplemental Figure 1A). Distal arterioles in these regions are reproducibly unmuscularized under basal conditions and become muscularized with hypoxia (10, 27–29). Muscularization of distal arterioles was quantified as the ratio of length of SMC coverage of the distal arteriole (from the middle-distal [M-D] arteriole border to the most distal SMA staining) to the entire length of the distal arteriole (from the M-D border to the capillary front; maximum distal arteriole length quantified was 300 μm).

Human lung samples from deidentified patients with IPAH and controls were obtained from the Pulmonary Hypertension Breakthrough Initiative (PHBI) (Supplemental Table 1). Frozen OCT-embedded tissues were sectioned, sections were fixed in 4% paraformaldehyde for 15 minutes at room temperature, washed 3 times in PBS, permeabilized with PBS-T for 10 minutes, and immunostained. Sections were incubated in primary antibodies diluted in blocking buffer overnight at 4°C, washed 3 times in PBS-T, and incubated in secondary antibodies for 1 hour at room temperature. Primary antibodies used were mouse anti-LC3B (1:100; Abcam, ab232940), mouse anti-Beclin1 (1:100; Abcam, ab114071), mouse anti-p62 (1:100; Abcam, ab56416), rabbit anti-ATG7 (1:100; Abcam, ab133528), rabbit anti-Cx40 (1:100; Abcam, ab213688), and/or anti-SMA directly conjugated to Cy3 or FITC (1:250; Sigma-Aldrich, C6198 or F3777, respectively). Secondary antibodies were conjugated to Alexa Fluor 488 and Alexa Fluor 568 (1:250; Invitrogen, A11029 or A11031). Nuclei were stained with DAPI (1:500).

### Murine lung EC and SMC isolation.

The lung vasculature was flushed with PBS as noted above, and lobes were carefully dissected from bronchi and mediastinal connective tissue. Lobes were finely minced and digested using the lung dissociation kit (Miltenyi Biotec, 130-095-927), mechanically dissociated with gentleMACS Dissociator (Miltenyi Biotec), filtered through a 70 μm cell strainer, and centrifuged for 5 minutes at 4°C. In preparation for isolation of ECs or SMCs by FACS and RNA analysis by qRT-PCR, the cell pellet was resuspended in 5% fetal bovine serum (FBS) in PBS to generate a single-cell suspension. For EC isolation, the single-cell suspension was incubated with anti-CD31–APC (1:200; BD Pharmingen, 551262) and anti-CD45–Pacific blue (1:200; Biolegend, 103126) antibodies for 30 minutes at 4°C. Cells were washed and resuspended in PBS with 1% FBS and propidium iodide (PI) was used as a viability dye (1:1000; Sigma-Aldrich, P4864). CD31^+^CD45^–^PI^–^ cells were sorted on a BD FACSAria II cell sorter. For SMC isolation, *Acta2-CreER^T2^* *ROSA26R^Zs/+^* mice were induced with tamoxifen (1 mg/day for 5 days), and a single-cell suspension was obtained. Cells were stained with PI, and FACS was used to isolate Zs^+^PI^–^cells. For Western blot analysis, lung ECs were isolated with anti-CD31–coated magnetic beads, as previously described (10). Briefly, sheep anti-rat-IgG Dynal magnetic beads (Invitrogen, 11035) were resuspended in PBS containing 0.1% FBS and incubated with rat anti-CD31 monoclonal antibody (1:250; BD Biosciences, 553370) overnight at 4°C. Beads were then washed and stored at 4°C. Lung single-cell suspensions in PBS containing 0.1% FBS were incubated with these anti-CD31–coated beads for 20 minutes at room temperature and washed 4 times with PBS. A magnet was used to separate cells bound to beads from unbound cells.

### Cell culture and hypoxia.

Human PASMCs and PAECs (American Type Culture Collection, PCS-100-023 and PCS-100-022, respectively) were cultured for up to 6 passages in SmGM 2 Smooth Muscle Cell Growth Medium-2 BulletKit (CC-3182) and EGM 2 Endothelial Cell Growth Medium-2 BulletKit (CC-3162) (both Lonza Bioscience), respectively. PASMCs were or were not treated with 20 ng/mL recombinant PDGF-B (Sigma-Aldrich) for 24 hours, and mRNA levels were determined by qRT-PCR. For mRNA decay assays, PASMCs were similarly cultured with PDGF-B or vehicle (DEPC water). Cells were then treated with 50 μmol/L of transcription inhibitor 5,6-dichloro-1-β-D-ribofuranosyl-benzimidazole (DRB; Sigma-Aldrich, D1916) and collected at 0, 1.5, 3, 6, 12, and 24 hours after initiation of DRB treatment for qRT-PCR analysis. For conditioned medium experiments, PAECs were subjected to either normoxia or 3% hypoxia for 6 or 16 hours at 37°C, and then, in select studies, treated with 20 μg/mL IgG isotype control or anti–PDGF-B blocking antibody (R&D Systems, AB-108-C or AF-220-NA, respectively) for 1 hour. The PAEC-conditioned medium was added to normoxic PASMCs for 48 hours. Cells were collected for qRT-PCR analysis.

### qRT-PCR analysis.

RNA from murine lung samples was isolated with the RNeasy Plus Kit (Qiagen) and reverse transcribed with the iScript cDNA Synthesis Kit (Bio-Rad). RNA lung samples from patients with IPAH and controls were obtained from the PHBI for qRT-PCR analysis (Supplemental Table 2). Transcript levels were determined by qRT-PCR and normalized to *18S*. Forward and reverse primer are listed in Supplemental Table 4.

### Western blot.

Lysates were prepared by mechanical homogenization of mouse lungs in lysis buffer (RIPA buffer and protease inhibitor cocktail) on ice with a glass pestle tissue homogenizer (Pyrex) or by suspension of EC-bound beads in lysis buffer. Lysates were centrifuged at 12,000*g* and 4°C for 5 minutes, supernatants were collected, and protein concentration was determined by BCA assay (Thermo Fisher Scientific). Protein samples were resolved by 7%–15% SDS-PAGE, transferred to Immobilon PVDF membranes (Millipore), blocked with 5% nonfat dry milk, washed in 0.1% Tween 20/TBS, and probed with primary antibodies overnight at 4°C. Membranes were incubated with HRP-conjugated secondary antibodies (Dako), washed in 0.1% Tween 20/TBS, developed with Supersignal West Pico Maximum Sensitivity Substrate (Pierce), and analyzed with the G:BOX imaging system (Syngene). Primary antibodies used for Western blot analysis were rabbit anti–HIF1-α (1:500; Novus, NB100-449), rabbit anti–HIF2-α (1:500; Novus, NB100-122), rabbit anti-Beclin1 (1:500; Novus, NB110-87318), rabbit anti-LC3B (1:500; Cell Signaling Technology, 2775), or rabbit anti-p62 (1:500; Abcam, ab109012).

### Imaging.

Lung sections were imaged on confocal microscopes (PerkinElmer UltraView VOX spinning disc or Leica SP5 point scanning). Volocity software (PerkinElmer) and Adobe Photoshop were used to process images.

### Statistics.

All data are presented as mean ± SD. Student’s *t* test and multifactor ANOVA with Tukey’s multiple-comparison test were used to analyze the data (GraphPad Prism software, v7). The statistical significance threshold was set at a *P* value of less than 0.05. All tests assumed normal distribution and were 2-sided.

### Study approval.

All mouse experiments were approved by the IACUC at Yale University. Deidentified human tissues were obtained from the PHBI in accordance with the Institutional Review Board of Yale University (IRB 1005006865). All experiments were performed in accordance with relevant ethical guidelines.

### Data and materials availability.

All data associated with this study are present in the paper or the supplemental information, and raw data are included in the Supporting Data Values file. Reagents and materials associated with this study are available from the corresponding author (DMG).

## Author contributions

All authors conceived of and designed experiments. FZS, AK, and JS performed them. All authors analyzed the results, prepared the figures, and wrote and revised the manuscript.

## Supplementary Material

Supplemental data

Unedited blot and gel images

Supporting data values

## Version 1 04/23/2024

In-Press Preview

## Version 2 05/22/2024

Electronic publication
