# Supplementary material for: Endothelial HIFα/PDGF-B to smooth muscle Beclin1 signaling sustains pathological muscularization in pulmonary hypertension
Source: JCI Insight. 2024 Apr 23;9(10):e162449. doi: 10.1172/jci.insight.162449 (PMC11141934; doi:10.1172/jci.insight.162449)
Supplement: Supplemental data [file jciinsight-9-162449-s058.pdf]

SUPPLEMENTAL INFORMATION

Pathological muscularization is maintained in pulmonary hypertension by signaling from endothelial HIFα-PDGF-B to smooth muscle Beclin1

Fatima Z. Saddouk, Andrew Kuzemczak, Junichi Saito, Daniel M. Greif

List of Supplemental Items:

- Supplemental Figures S1-S15
- Supplemental Legends S1-S15
- Supplemental Tables S1-S4

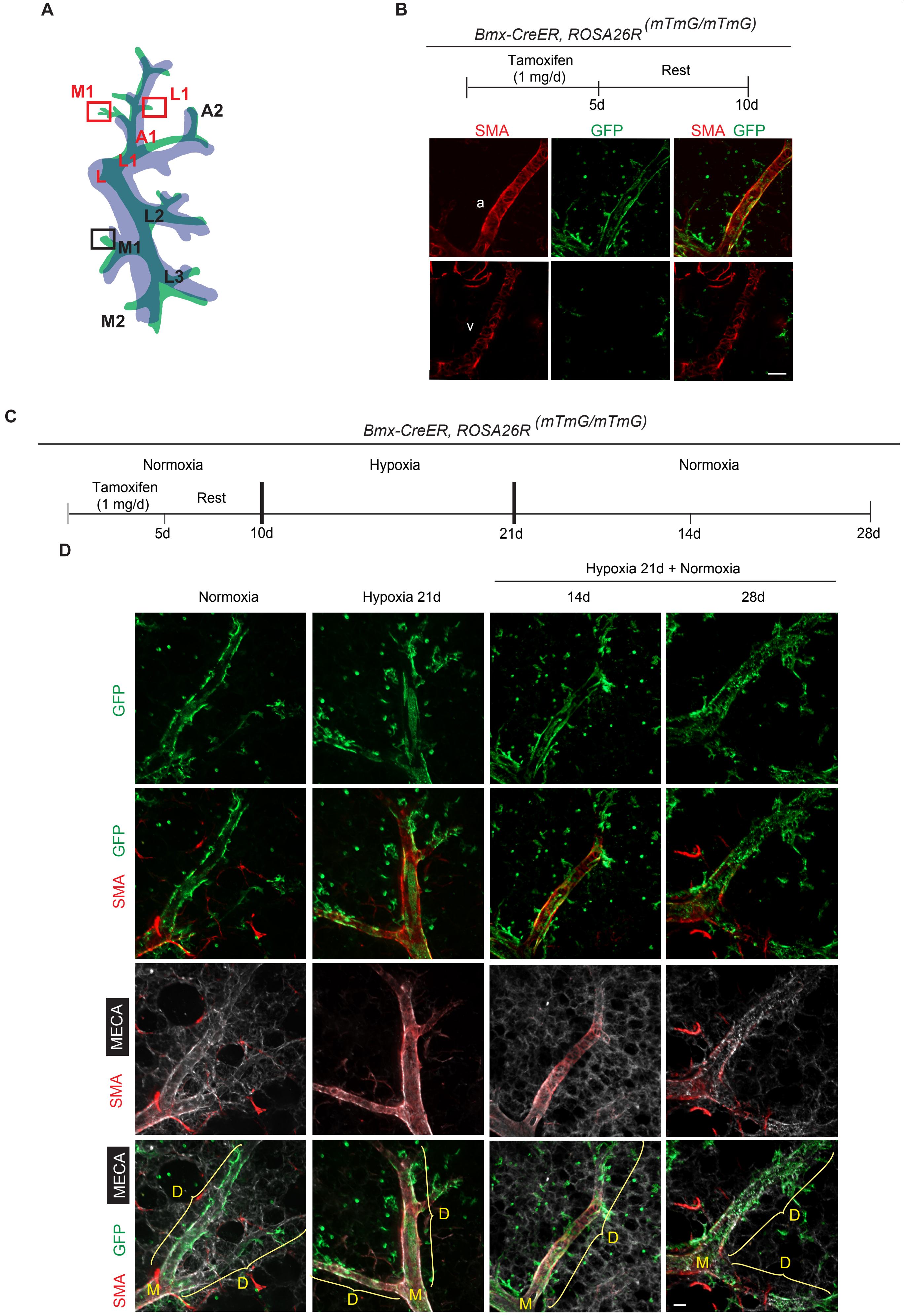
A
M1
L1
A2
A1
L1
L
L2
M1
L3
M2
B
Bmx-CreER, ROSA26R (mTmG/mTmG)
Tamoxifen (1 mg/d)
Rest
5d
10d
SMA
GFP
SMA GFP
a
v
C
Bmx-CreER, ROSA26R (mTmG/mTmG)
Normoxia
Hypoxia
Normoxia
Tamoxifen (1 mg/d)
Rest
5d
10d
21d
14d
28d
D
Hypoxia 21d + Normoxia
Normoxia
Hypoxia 21d
14d
28d
GFP
SMA GFP
SMA MECA
SMA GFP MECA
D
M

Saddouk et al., Fig. S2

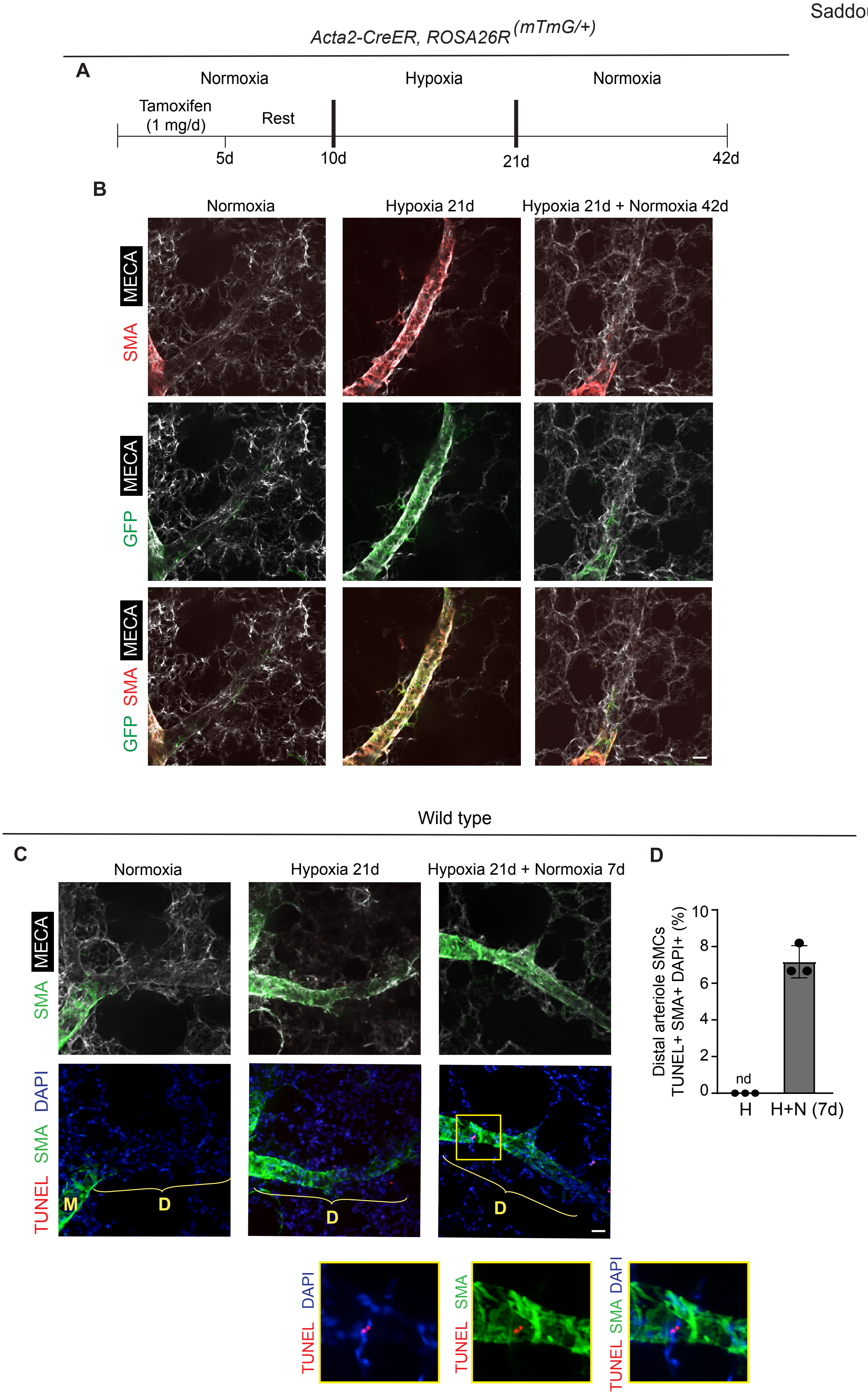

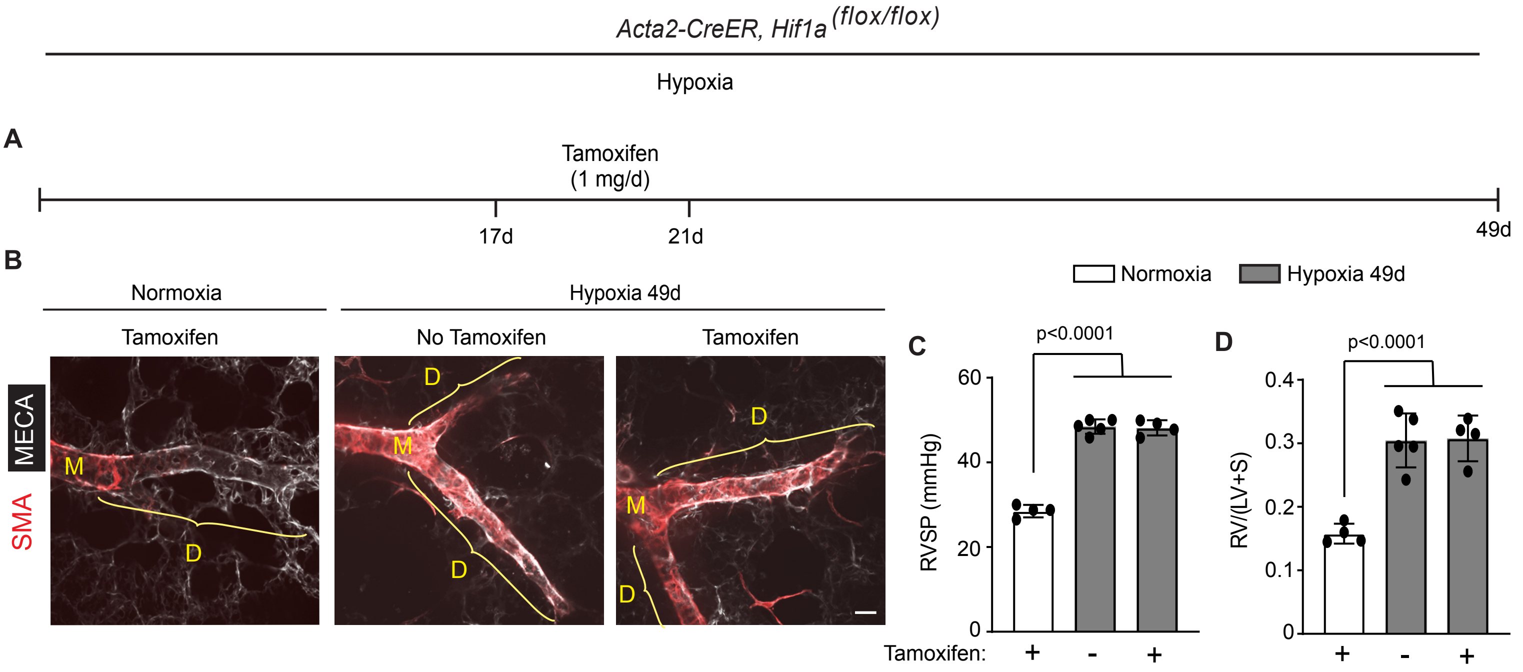
Acta2-CreER, Hif1a (flox/flox)
Hypoxia
A
Tamoxifen
(1 mg/d)
17d
21d
49d
B
Normoxia
Hypoxia 49d
Tamoxifen
No Tamoxifen
Tamoxifen
MECA
SMA
M
D
Normoxia
Hypoxia 49d
C
p<0.0001
60
40
20
0
RVSP (mmHg)
Tamoxifen:
+
-
+
D
p<0.0001
0.4
0.3
0.2
0.1
0
RV/(LV+S)
+
-
+

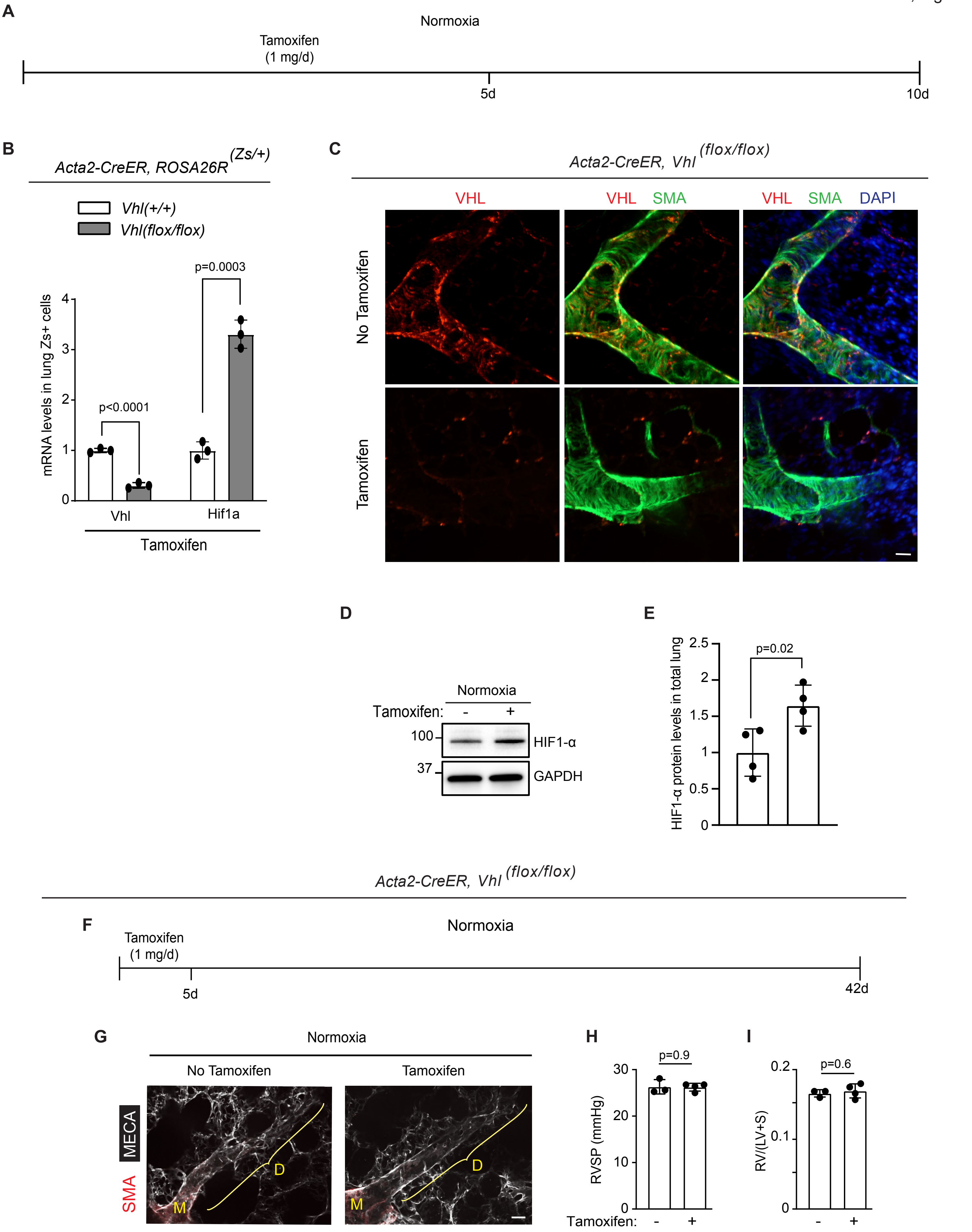
A
Normoxia
Tamoxifen
(1 mg/d)
5d
10d
B
Acta2-CreER, ROSA26R(Zs/+)
Vhl(+/+)
Vhl(flox/flox)
p=0.0003
p<0.0001
mRNA levels in lung Zs+ cells
0
1
2
3
4
Vhl
Hif1a
Tamoxifen
C
Acta2-CreER, Vhl(flox/flox)
VHL
VHL SMA
VHL SMA DAPI
No Tamoxifen
Tamoxifen
D
Normoxia
Tamoxifen: - +
100
37
HIF1-α
GAPDH
E
p=0.02
HIF1-α protein levels in total lung
0
0.5
1
1.5
2
2.5
Acta2-CreER, Vhl(flox/flox)
F
Normoxia
Tamoxifen
(1 mg/d)
5d
42d
G
Normoxia
No Tamoxifen
Tamoxifen
MECA
SMA
M
D
H
p=0.9
RVSP (mmHg)
0
10
20
30
Tamoxifen: - +
I
p=0.6
RV/(LV+S)
0
0.1
0.2
- +

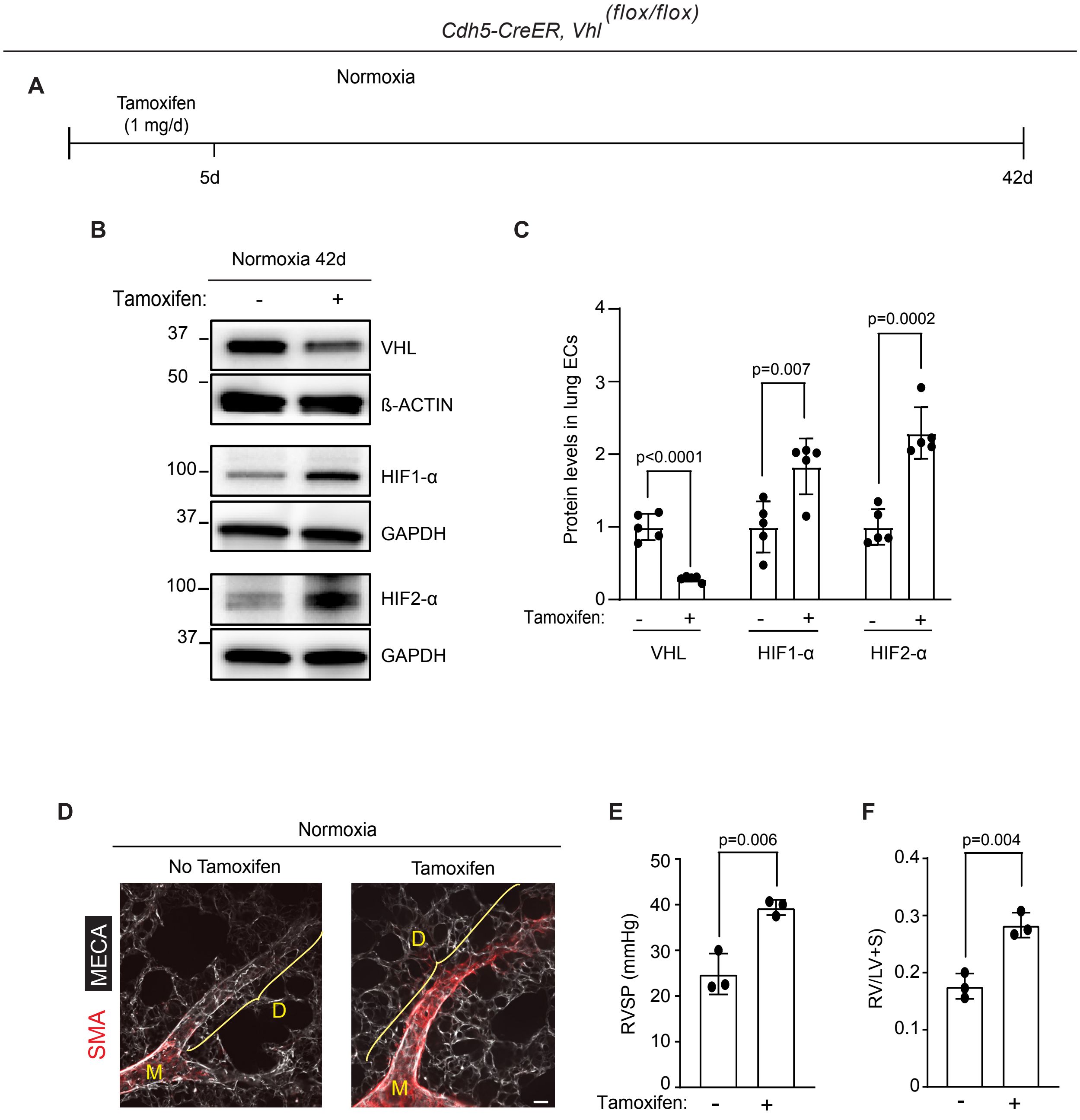
Cdh5-CreER, Vhl (flox/flox)
A
Normoxia
Tamoxifen
(1 mg/d)
5d
42d
B
Normoxia 42d
Tamoxifen: - +
37
50
VHL
ß-ACTIN
100
HIF1-α
37
GAPDH
100
HIF2-α
37
GAPDH
C
Protein levels in lung ECs
p<0.0001
p=0.007
p=0.0002
Tamoxifen: - + - + - +
VHL
HIF1-α
HIF2-α
D
Normoxia
No Tamoxifen
Tamoxifen
MECA
SMA
D
M
E
p=0.006
RVSP (mmHg)
Tamoxifen: - +
F
p=0.004
RV/LV+S)

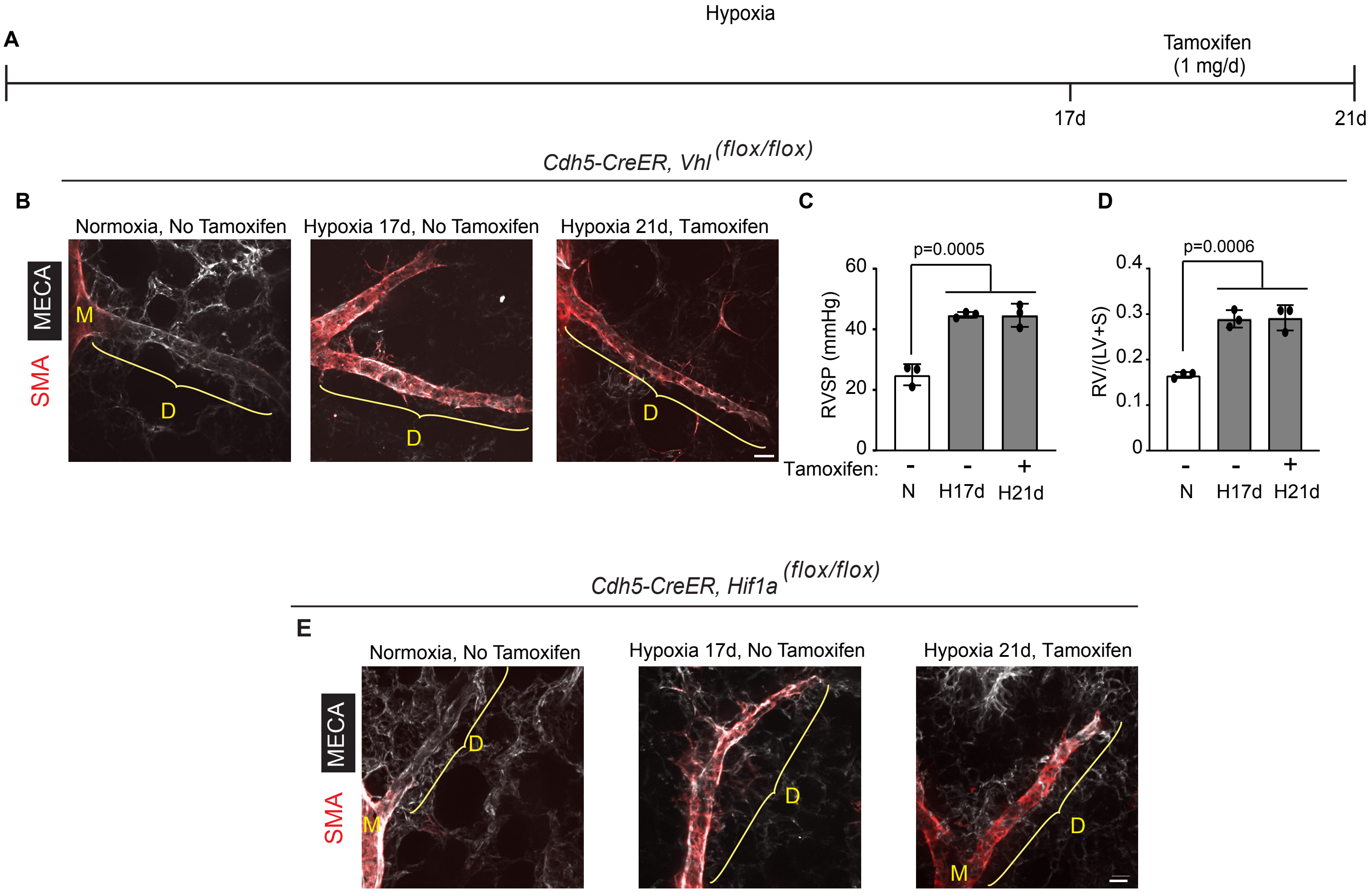
Hypoxia
A
Tamoxifen
(1 mg/d)
17d
21d
Cdh5-CreER, Vhl (flox/flox)
B
Normoxia, No Tamoxifen
Hypoxia 17d, No Tamoxifen
Hypoxia 21d, Tamoxifen
MECA
SMA
M
D
C
p=0.0005
RVSP (mmHg)
60
40
20
0
Tamoxifen:
-
-
+
N
H17d
H21d
D
p=0.0006
RV/(LV+S)
0.4
0.3
0.2
0.1
0
-
-
+
N
H17d
H21d
Cdh5-CreER, Hif1a (flox/flox)
E
Normoxia, No Tamoxifen
Hypoxia 17d, No Tamoxifen
Hypoxia 21d, Tamoxifen
MECA
SMA
M
D

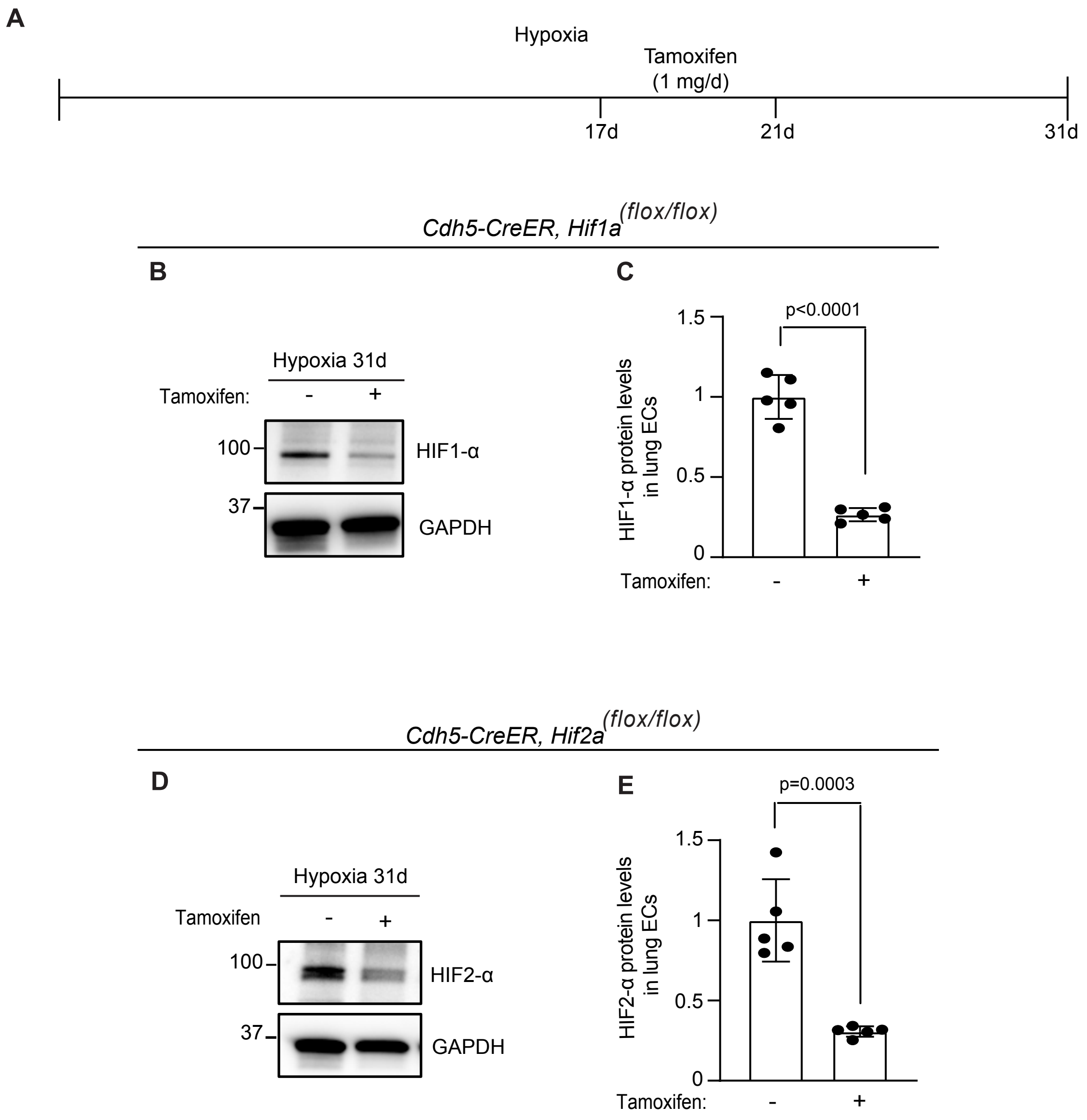
A
Hypoxia
Tamoxifen
(1 mg/d)
17d
21d
31d
Cdh5-CreER, Hif1a(flox/flox)
B
Hypoxia 31d
Tamoxifen:
-
+
100
HIF1-α
37
GAPDH
C
p<0.0001
1.5
1
0.5
0
HIF1-α protein levels in lung ECs
Tamoxifen:
-
+
Cdh5-CreER, Hif2a(flox/flox)
D
Hypoxia 31d
Tamoxifen
-
+
100
HIF2-α
37
GAPDH
E
p=0.0003
1.5
1
0.5
0
HIF2-α protein levels in lung ECs
Tamoxifen:
-
+

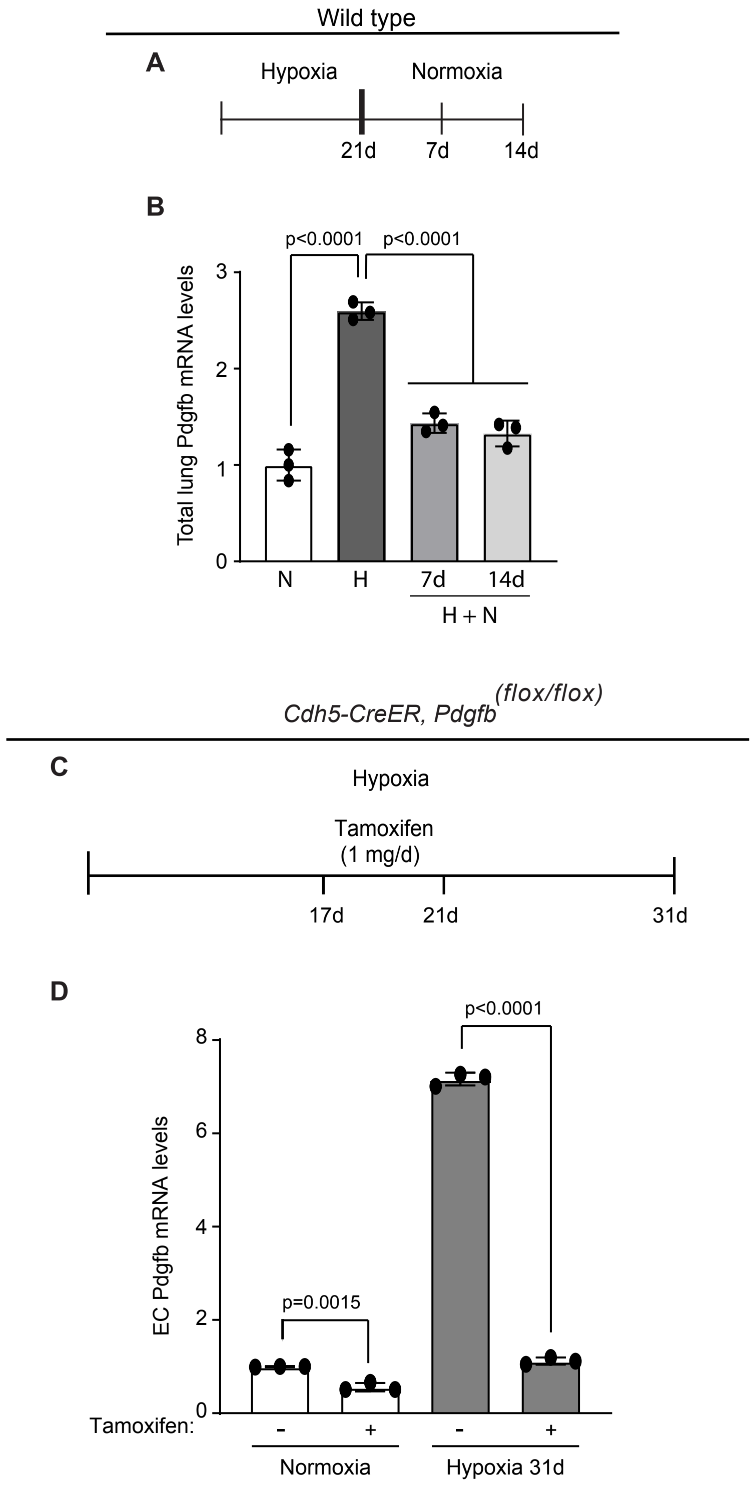
Wild type
A
Hypoxia
Normoxia
21d
7d
14d
B
p<0.0001
p<0.0001
Total lung Pdgfb mRNA levels
3
2
1
0
N
H
7d
14d
H + N
Cdh5-CreER, Pdgfb(flox/flox)
C
Hypoxia
Tamoxifen
(1 mg/d)
17d
21d
31d
D
p<0.0001
p=0.0015
EC Pdgfb mRNA levels
8
6
4
2
0
Tamoxifen:
-
+
-
+
Normoxia
Hypoxia 31d

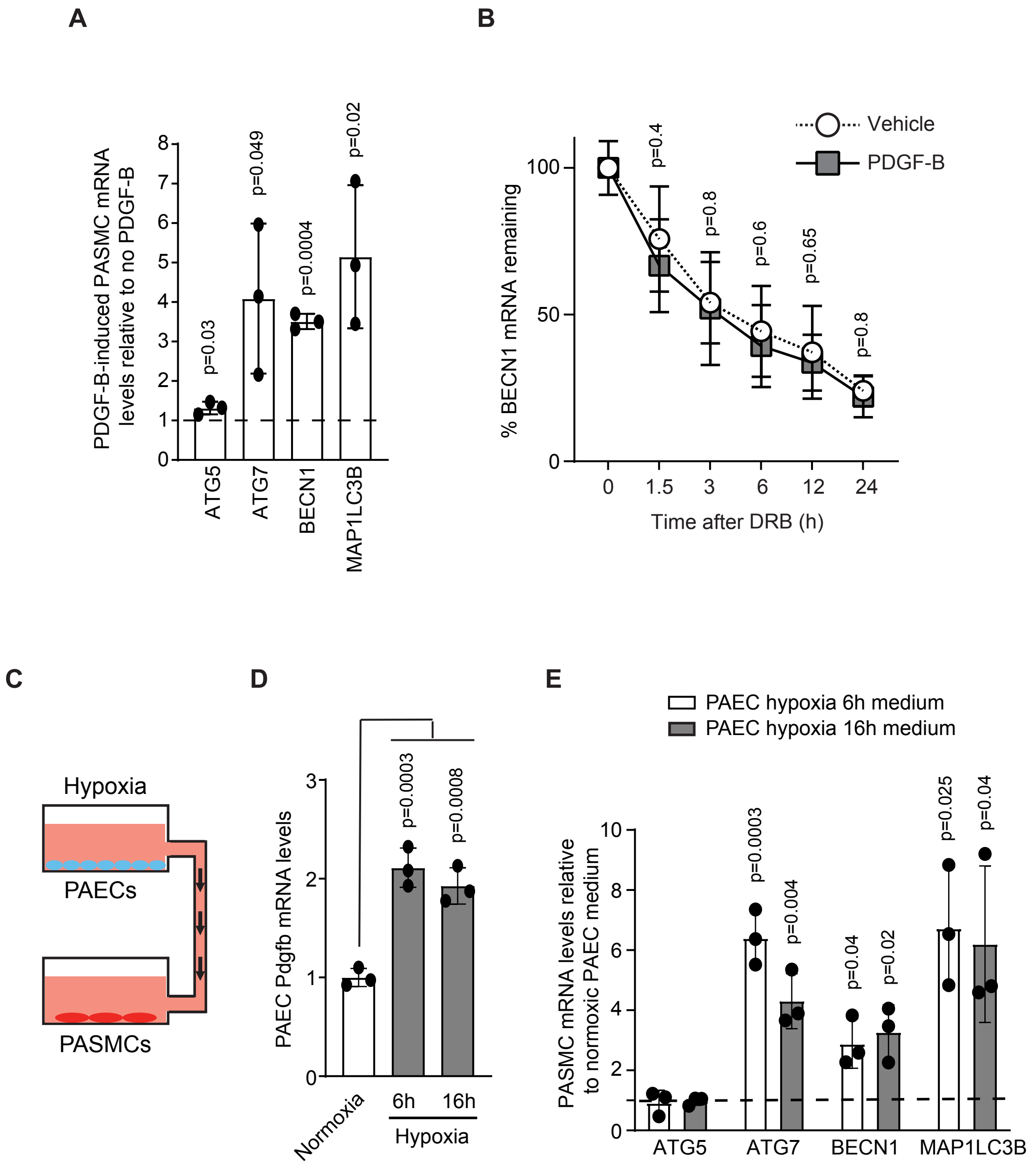
A
PDF-B-induced PASMC mRNA levels relative to no PDGF-B
p=0.03
p=0.049
p=0.0004
p=0.02
ATG5
ATG7
BECN1
MAP1LC3B
B
Vehicle
PDGF-B
% BECN1 mRNA remaining
p=0.4
p=0.8
p=0.6
p=0.65
p=0.8
Time after DRB (h)
C
Hypoxia
PAECs
PASMCs
D
PAEC Pdgfb mRNA levels
p=0.0003
p=0.0008
Normoxia
6h
16h
Hypoxia
E
PAEC hypoxia 6h medium
PAEC hypoxia 16h medium
PASMC mRNA levels relative to normoxic PAEC medium
p=0.0003
p=0.004
p=0.04
p=0.02
p=0.025
p=0.04
ATG5
ATG7
BECN1
MAP1LC3B

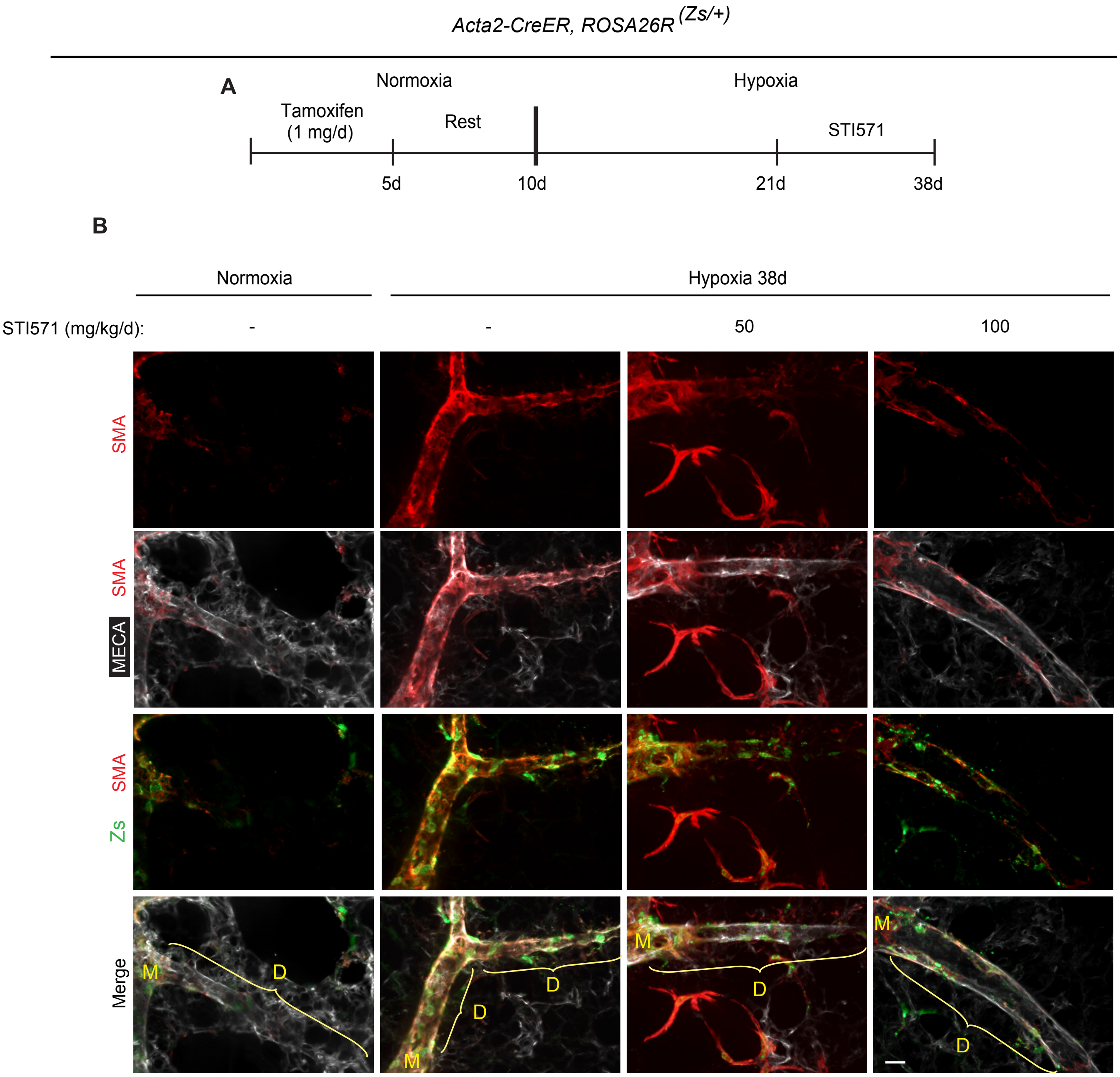
Acta2-CreER, ROSA26R (Zs/+)
A
Normoxia
Hypoxia
Tamoxifen (1 mg/d)
Rest
STI571
5d
10d
21d
38d
B
Normoxia
Hypoxia 38d
STI571 (mg/kg/d):
-
-
50
100
SMA
SMA
MECA
SMA
Zs
Merge
M
D

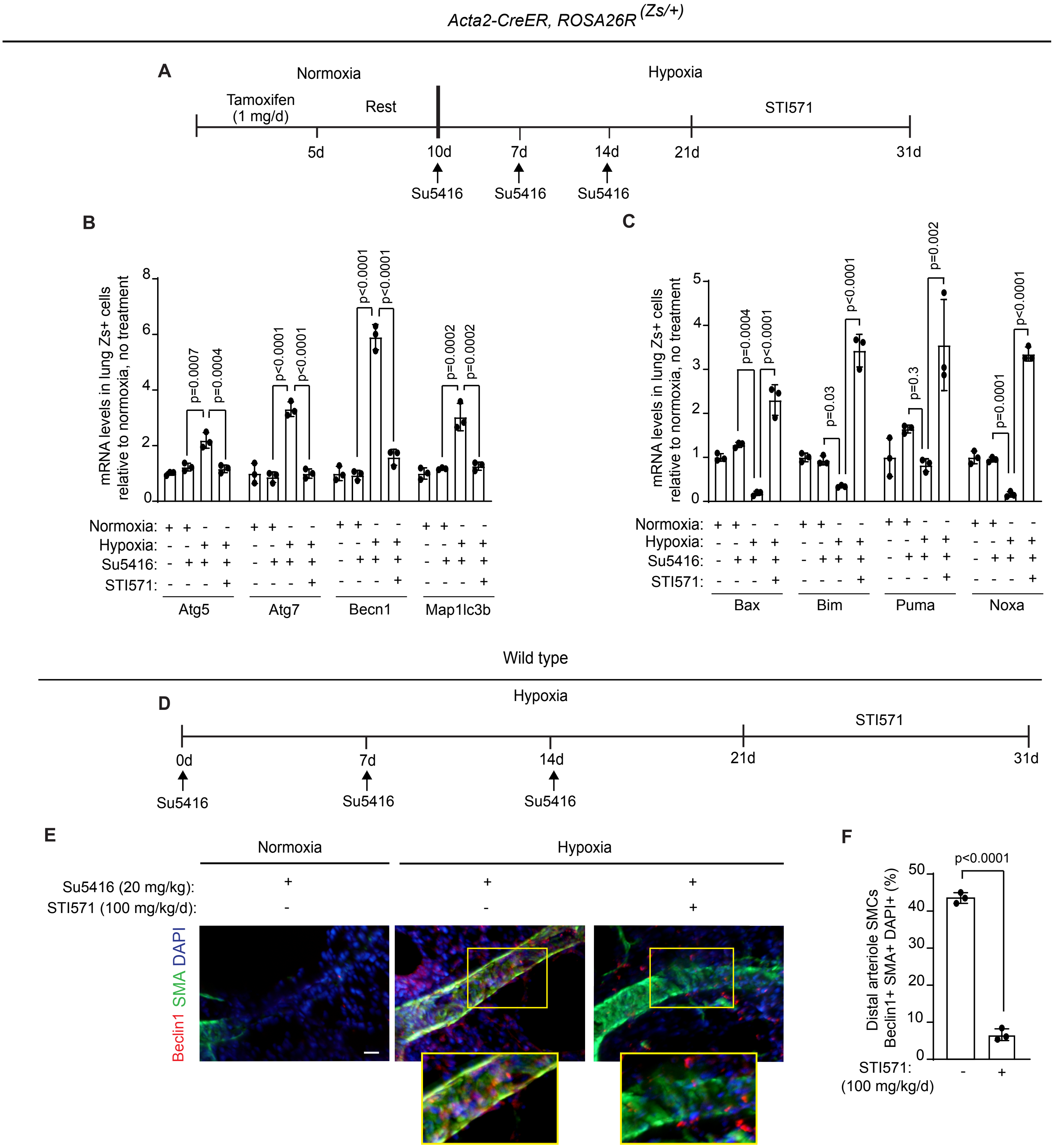

Acta2-CreER, ROSA26R (Zs/+)
A
Normoxia
Hypoxia
Tamoxifen (1 mg/d)
Rest
STI571
5d
10d
7d
14d
21d
31d
Su5416
Su5416
Su5416
B
mRNA levels in lung Zs+ cells relative to normoxia, no treatment
p=0.0007
p=0.0004
p<0.0001
p<0.0001
p<0.0001
p<0.0001
p=0.0002
p=0.0002
Normoxia:
Hypoxia:
Su5416:
STI571:
Atg5
Atg7
Becn1
Map1lc3b
C
p=0.0004
p<0.0001
p=0.03
p<0.0001
p=0.3
p=0.002
p=0.0001
p<0.0001
Bax
Bim
Puma
Noxa
Wild type
D
Hypoxia
STI571
0d
7d
14d
21d
31d
Su5416
Su5416
Su5416
E
Normoxia
Hypoxia
Su5416 (20 mg/kg):
STI571 (100 mg/kg/d):
Beclin1 SMA DAPI
F
p<0.0001
Distal arteriole SMCs Beclin1+ SMA+ DAPI+ (%)
STI571: (100 mg/kg/d)

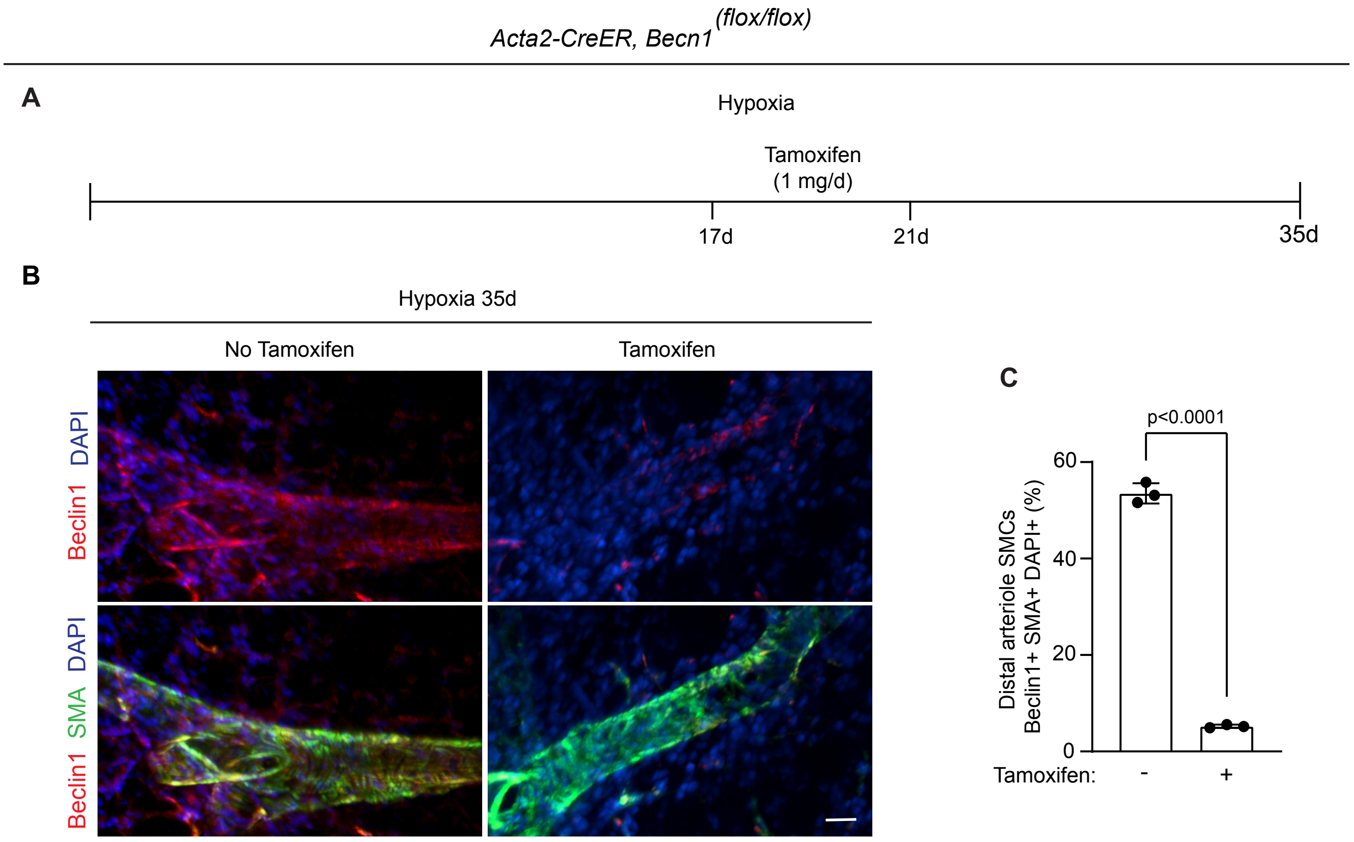

Acta2-CreER, Becn1 (flox/flox)
A
Hypoxia
Tamoxifen
(1 mg/d)
17d
21d
35d
B
Hypoxia 35d
No Tamoxifen
Tamoxifen
Beclin1 DAPI
Beclin1 SMA DAPI
C
p<0.0001
60
40
20
0
Distal arteriole SMCs
Beclin1+ SMA+ DAPI+ (%)
Tamoxifen:
-
+

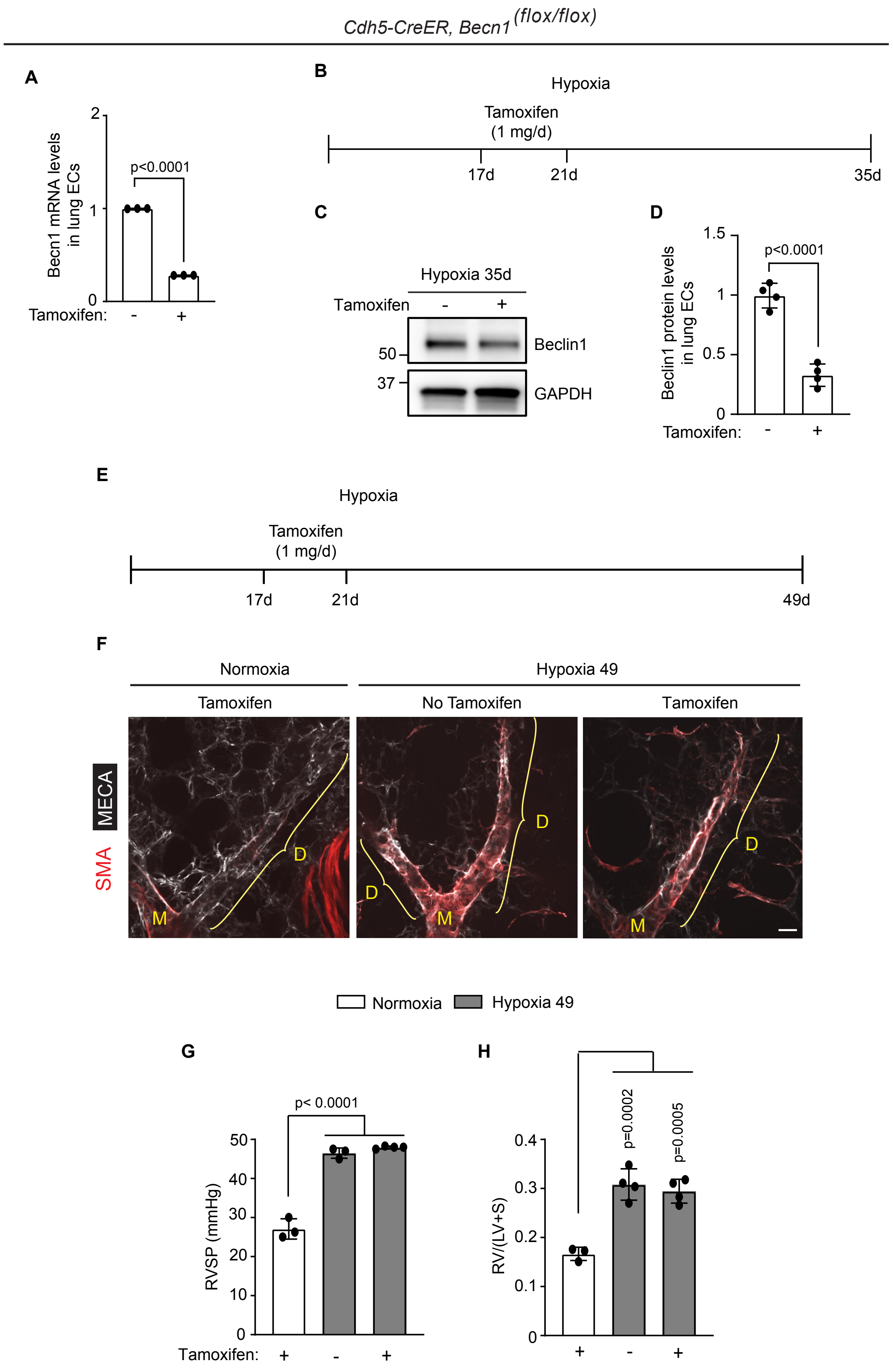
Cdh5-CreER, Becn1 (flox/flox)
A
Becn1 mRNA levels in lung ECs
2
1
0
p<0.0001
Tamoxifen: - +
B
Hypoxia
Tamoxifen (1 mg/d)
17d
21d
35d
C
Hypoxia 35d
Tamoxifen - +
50
37
Beclin1
GAPDH
D
Beclin1 protein levels in lung ECs
1.5
1
0.5
0
p<0.0001
Tamoxifen: - +
E
Hypoxia
Tamoxifen (1 mg/d)
17d
21d
49d
F
Normoxia
Hypoxia 49
Tamoxifen
No Tamoxifen
Tamoxifen
MECA
SMA
D
M
Normoxia
Hypoxia 49
G
RVSP (mmHg)
50
40
30
20
10
0
p< 0.0001
Tamoxifen: + - +
H
RV/(LV+S)
0.4
0.3
0.2
0.1
0
p=0.0002
p=0.0005
+ - +

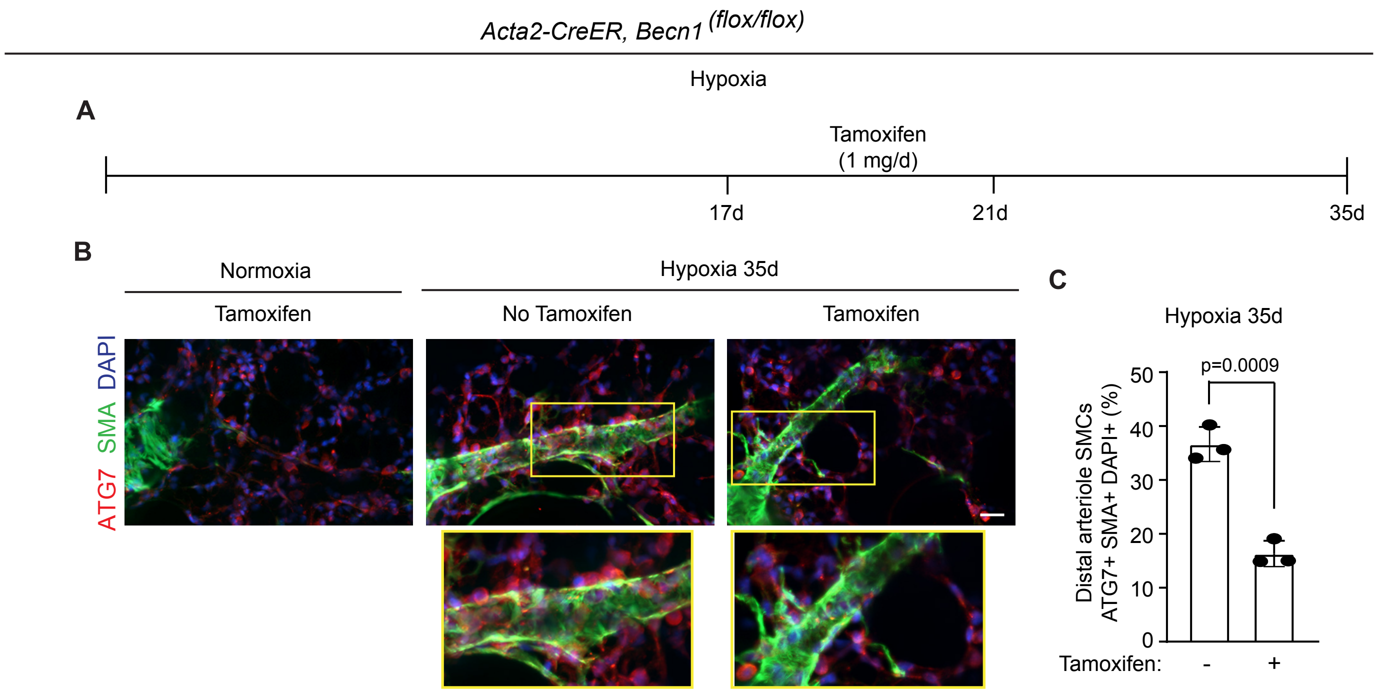

Acta2-CreER, Becn1 (flox/flox)
Hypoxia
A
Tamoxifen
(1 mg/d)
17d
21d
35d
B
Normoxia
Hypoxia 35d
Tamoxifen
No Tamoxifen
Tamoxifen
ATG7 SMA DAPI
C
Hypoxia 35d
p=0.0009
Distal arteriole SMCs
ATG7+ SMA+ DAPI+ (%)
0
10
20
30
40
50
Tamoxifen:
-
+

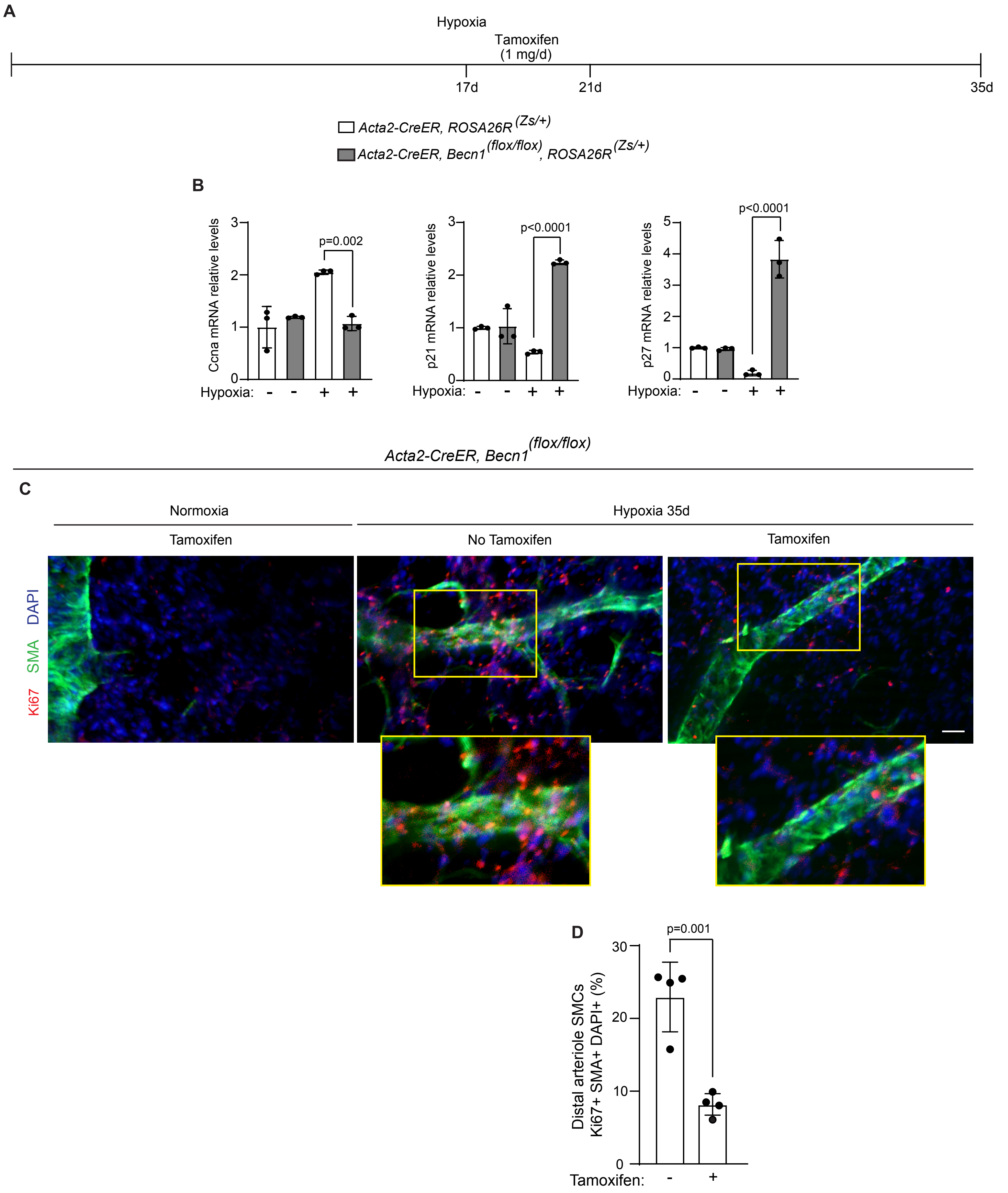
A
Hypoxia
Tamoxifen
(1 mg/d)
17d
21d
35d
Acta2-CreER, ROSA26R (Zs/+)
Acta2-CreER, Becn1 (flox/flox), ROSA26R (Zs/+)
B
Ccna mRNA relative levels
p=0.002
p21 mRNA relative levels
p<0.0001
p27 mRNA relative levels
p<0.0001
Hypoxia: - - + +
Acta2-CreER, Becn1 (flox/flox)
C
Normoxia
Hypoxia 35d
Tamoxifen
No Tamoxifen
Tamoxifen
DAPI
SMA
Ki67
D
p=0.001
Distal arteriole SMCs Ki67+ SMA+ DAPI+ (%)
Tamoxifen: - +

Supplementary Figure Legends

Figure S1. Reversal of specific distal pulmonary arteriole muscularization with re-normoxia following hypoxia. A, Schematic of left lung proximal artery (green) and airway (blue) branches. Boxes identify regions where distal arteriole beds are reproducibly unmuscularized under basal conditions and become muscularized with hypoxia. The red boxes specifically indicate the arteriole beds shown in (D). These arteriole beds are in proximity to airway branches L.L1.A1.L1 and L.L1.A1.M1 (i.e., left bronchus-first lateral secondary branch-first anterior branch-first lateral branch). L, left main bronchus; L1, L2, L3, lateral branches; M1, M2, medial branches; A1, anterior branch. **B,** *Bmx-CreER^{T2}, ROSA26R$^{(mTmG/mTmG)}$* mice were induced with tamoxifen (1 mg/day for 5 days) and then rested for 5 days. Vibratome lung sections were stained for SMA and GFP. n=3 mice (1 male, 2 females). Pulmonary arteries (a) have a continuous layer of SMA-stained SMCs whereas pulmonary veins (v) are coated by irregular SMA^{+} cells, forming a lose mesh around the vessel. **C,** Experimental strategy for (D). **D,** *Bmx-CreER^{T2}, ROSA26R$^{(mTmG/mTmG)}$* mice were induced with tamoxifen (1 mg/day for 5 days), rested, exposed to hypoxia for 21 days and then immediately analyzed or re-exposed to normoxia for 14 to 28 days. Lung vibratome sections were stained for GFP, SMA and MECA-32. n=3 mice (1 male, 2 females) per experimental group, 2 arterioles analyzed per mouse. M and D, middle and distal arterioles are denoted, respectively. Scale bars, 50 µm (B) and 20 µm (D).

Figure S2. Distal arteriole SMCs undergo apoptosis, not fate change, during re-exposure to normoxia. A, Experimental strategy for (B). **B,** *Acta2-CreER^{T2}, ROSA26R$^{(mTmG/+)}$* mice were injected with tamoxifen (1 mg/day for 5 days), rested, exposed to hypoxia for 21 days and then immediately analyzed or re-exposed to normoxia for 42 days. Lung vibratome sections were

stained for GFP, SMA and MECA-32. n=4 mice (2 males, 2 females) per experimental group, 3 arterioles analyzed per mouse. **C,** Wild type mice were exposed to normoxia or hypoxia for 21 days and immediately analyzed or exposed to 7 days of normoxia prior to analysis. Vibratome lung sections were stained for TUNEL, SMA, MECA-32 and nuclei (DAPI) with close-ups of boxed region shown below. M and D, middle and distal pulmonary arterioles, respectively. **D,** Quantification of the percent of SMCs that are $TUNEL^{+}$ in distal arterioles. n=3 mice (2 males, 1 female) per experimental group, 2-3 arteriole analyzed per lung. nd, not detected. Scale bars, 20 μm.

Figure S3. In SMCs, *Hif1a* deletion does not reverse established distal arteriole muscularization and PH. A, Experimental strategy for (B-D). **B,** *Acta2-CreER*^{T2}, *Hif1a*$^{(flox/flox)}$ mice were exposed to hypoxia for 49 days and tamoxifen (1 mg/day) was or was not administered between hypoxia days 17-21. Vibratome lung sections were stained for SMA and MECA-32. **C, D,** RVSP and the RV weight ratio were measured. n=4-5 mice (2 males, 2-3 females) per experimental group, 3 arterioles analyzed per mouse. Scale bar, 20 μm.

Figure S4. In SMCs, *Vhl* deletion does not induce new pulmonary vascular remodeling. A, Experimental strategy for (B-E). **B,** *Acta2-CreER*^{T2}, *ROSA26R*$^{(Zs/+)}$ mice carrying *Vhl*$^{(+/+)}$ or *Vhl*$^{(flox/flox)}$ were induced with tamoxifen and then rested for 5 days, and Zs^{+} cells were isolated by FACS. *Vhl* and *Hif1a* transcript levels in Zs^{+} cells were measured with qRT-PCR. n=3 mice (1 male, 2 females) per experimental group. **C-E,** *Acta2-CreER*^{T2}, *Vhl*$^{(flox/flox)}$ were or were not induced with tamoxifen and then were rested for 5 days. In (C), vibratome lung sections were stained for SMA and VHL. n=4 mice (2 males, 2 females) per experimental group, 3 arterioles

analyzed per mouse. In (D, E), Western blots of whole lung lysates probed for HIF1α and GAPDH, and densitometry of protein bands relative to GAPDH and normalized to no tamoxifen treatment. n=4 mice (2 males, 2 females) per experimental group. **F,** Experimental strategy for (G-I). **G,** Under normoxia, *Acta2-CreER*^{T2}*, Vhl*$^{(flox/flox)}$ mice were or not injected with tamoxifen (1 mg/day for 5 days) and then rested for 37 days. Lung vibratome sections were stained for SMA and MECA-32. **H, I,** RVSP and RV weight ratio were measured. n=3-4 mice (1-2 males, 2 females) per experimental group, 2 arterioles analyzed per mouse. M and D, middle and distal arterioles, respectively. Scale bars, 20 μm.

Figure S5. Tamoxifen treatment of *Cdh5-CreER*^{T2}*, Vhl*$^{(flox/flox)}$ mice induces distal arteriole muscularization and PH under normoxia. A, Experimental strategy for (B-F). **B, C,** Under normoxia, *Cdh5-CreER*^{T2}*, Vhl*$^{(flox/flox)}$ were or not injected with tamoxifen (1 mg/day for 5 days) and then rested for 37 days. ECs were isolated from murine lungs using anti-CD31-coated beads and subjected to Western blot analysis. Lysates probed for VHL, HIF1-α, HIF2-α, β-Actin and GAPDH are shown (B) with densitometry of protein bands (C) relative to β-Actin or GAPDH and normalized to no tamoxifen. n=5 mice (2 males, 3 females) per experimental group. **D,** Lung vibratome sections were stained for SMA and MECA-32. **E, F,** RVSP and the RV weight ratio were measured. n=3 mice (2 males, 1 female) per experimental group, 3 arterioles per mouse. Scale bar, 20 μm.

Figure S6. Tamoxifen treatment of *Cdh5-CreER*^{T2} mice carrying *Vhl*$^{(flox/flox)}$ or *Hif1a*$^{(flox/flox)}$ between hypoxia days 17-21 does not alter pulmonary vascular remodeling. A, Experimental strategy for (B-E). **B-E,** *Cdh5-CreER*^{T2} mice carrying *Vhl*$^{(flox/flox)}$ (B-D) or *Hif1a*$^{(flox/flox)}$ (E) were

exposed to hypoxia for 21 days and tamoxifen (1 mg/day) was or was not administered between hypoxia days 17-21. Lungs were collected at days 17 and 21. Vibratome lung sections were stained for SMA and MECA-32. In (C, D), RVSP and the RV weight ratio were measured. n=3 mice (1 male, 2 females) per experimental group, 3 arterioles per mouse. Scale bars, 20 µm.

Figure S7: Efficiency of *Hif1a* and *Hif2a* deletion in ECs. A, Experimental strategy for (B-E). **B, D**, *Cdh5-CreER*^{T2} mice carrying *Hif1a*$^{(flox/flox)}$ or *Hif2a*$^{(flox/flox)}$ were treated with hypoxia for 31 days and tamoxifen (1 mg/day) was or was not administered between hypoxia days 17-21. At 31 days, lung ECs were isolated using anti-CD31-coated beads and subjected to Western blot analysis. EC lysates were probed for HIF1α, HIF2α and GAPDH. **C, E**, Densitometry of protein bands shown in (B, D), respectively relative to GAPDH and normalized to no tamoxifen. n=5 mice (2 males, 3 females) per experimental group.

Figure S8. Lung Pdgfb levels are downregulated during re-normoxia and efficiency of genetic *Pdgfb* deletion in ECs. A, Experimental strategy for (B). **B,** Wild type mice were exposed to normoxia or hypoxia for 21 days followed by normoxia for 7 or 14 days. Lungs were harvested for RNA analysis. *Pdgfb* transcript levels were measured with qRT-PCR. n=3 mice (2 males, 1 female) per experimental group. **C,** Experimental strategy for (D). **D,** *Cdh5-CreER*^{T2}*, Pdgfb*$^{(flox/flox)}$ mice were exposed to hypoxia for 31 days and tamoxifen (1 mg/day) was or was not administered between hypoxia days 17-21. At hypoxia day 31, lung $CD31^{+}CD45^{-}$ ECs were isolated by FACS. qRT-PCR was used to measure *Pdgfb* transcript level. n=3 mice (1 males, 2 females) per experimental group.

Figure S9. Hypoxia-induced PDGF-B from human PAECs regulates autophagy-related genes in cultured human PASMCs. A, B, Human PASMCs were cultured with PDGF-B (20 ng/ml) for 24 hours. In (A), mRNA levels of autophagy-related genes were measured by qRT-PCR in cells cultured with, relative to without, PDGF-B. In (B), following culturing with PDGF-B or vehicle, cells were treated with the transcription inhibitor DRB (50 μmol/L) for the indicated times, at which point Becn1 transcript levels were determined. **C,** Experimental strategy for (D, E). **D,** Human PAECs were exposed to hypoxia (3% O_2) for 6 or 16 h, and EC Pdgfb transcript levels was measured with qRT-PCR. n=3. **E,** Human PAECs were exposed to hypoxia (3% O_2) for 6 or 16 h and then the medium was collected and added to human PASMCs under normoxic conditions for 48 h. qRT-PCR was used to assess PASMC autophagy-related gene transcript levels (n=3). Transcript levels relative to 18S rRNA were normalized to treatments with no PDGF-B in (A) or normoxic PAEC medium in (E).

Figure S10. STI571 treatment reverses established distal arteriole muscularization. **A.** Experimental strategy for (B). **B,** *Acta2-CreER^{T2}, ROSA26R$^{(Zs/+)}$* mice were induced with tamoxifen (1 mg/day for 5 days), rested and then exposed to hypoxia or normoxia for 38 days and STI571(0, 50 or 100 mg/kg/d) was administered by daily intraperitoneal injections between hypoxia days 21-38. Vibratome lung sections were stained for SMA, Zs and MECA-32. n=3 mice (1 male, 2 females) per experimental group, 3 arterioles per mouse. Scale bar, 20 μm.

Figure S11. STI571 treatment in the Sugen 5416/hypoxia model downregulates Beclin1 and upregulates apoptosis markers. **A,** Experimental strategy for (B, C). **B, C,** *Acta2-CreER^{T2}, ROSA26R$^{(Zs/+)}$* mice were induced with tamoxifen (1 mg/day for 5 days), rested and then exposed

to hypoxia or normoxia for 31 days. Sugen 5416 (20 mg/kg/dose) was injected subcutaneously at hypoxia days 0, 7 and 14, and STI571 (0 or 100 mg/kg/d) was administered by daily intraperitoneal injections between hypoxia days 21-31. Lung Zs^{+} SMCs were isolated by FACS, and expression levels of autophagy markers in (B) and apoptosis markers in (C) with hypoxia relative to normoxia, no STI571 were analyzed by qRT-PCR. n=3 mice (2 males, 1 female) per experimental group. **D,** Experimental strategy for (E, F). **E**, Wild type mice were exposed to hypoxia or normoxia for 31 days. Sugen 5416 (20 mg/kg/dose) was subcutaneously injected at days 0, 7, 14, and STI571 (0 or 100 mg/kg/d) was administered intraperitoneally each of the days 21-38. Vibratome lung sections were stained for Beclin1, SMA and nuclei (DAPI). Close-ups of boxed regions regions are shown below. **F**, Quantification of the percent of distal arteriole SMCs that are Beclin1^{+}. n=3 mice (2 males, 1 female) per experimental group, 3-4 arterioles analyzed per mouse. Scale bar, 20 µm.

Figure S12. Efficiency of *Becn1* deletion in distal pulmonary arteriole SMCs. A, Experimental strategy for (B, C). **B,** *Acta2-CreER^{T2}, Becn1$^{(flox/flox)}$* mice were treated with hypoxia for 35 days, and tamoxifen was or was not administered between hypoxia days 17-21. Vibratome lung sections were stained for Beclin1, SMA and nuclei (DAPI). **C**, Quantification of the percent of distal arteriole SMCs that are Belclin1^{+}. n=3 mice (2 males, 1 female) per experimental group, 3 arterioles analyzed per mouse. Scale bar, 20 µm.

Figure S13. EC deletion of *Becn1* does not alter established distal arteriole muscularization and PH. *Cdh5-CreER^{T2}, Becn1$^{(flox/flox)}$* mice were used in these studies. **A,** Mice were induced with tamoxifen (1 mg/day for 5 days), rested for 5 days and lung CD31^{+}CD45^{-} ECs were isolated

by FACS. *Becn1* transcript levels were measured by qRT-PCR. n=3 mice (2 males, 1 female) per experimental group. **B,** Experimental strategy for (C, D). **C,** Mice were treated with hypoxia for 35 days, and tamoxifen was or was not administered between hypoxia days 17-21, and lung ECs were isolated using anti-CD31-coated beads. EC lysates were analyzed by Western blotting for Beclin1 and GAPDH. **D,** Densitometry of protein bands shown in (C) relative to GAPDH and normalized to no tamoxifen treatment. n=4 mice (2 males, 2 females) per experimental group. **E,** Experimental strategy for (F-H). Mice were exposed to hypoxia for 49 days and tamoxifen (1 mg/day) was or was not administered between hypoxia days 17-21. **F,** Vibratome lung sections were stained for SMA and MECA-32. **G, H,** RVSP and RV weight ratio were measured. n=3-4 mice (2 males, 1-2 females) per experimental group, 3 arterioles analyzed per mouse. Scale bar, 20 μm.

Figure S14. SMC deletion of *Becn1* attenuates autophagy marker ATG7 expression. A, Experimental strategy for (B, C). **B,** *Acta2-CreER^{T2}, Becn1$^{(flox/flox)}$* mice were exposed to hypoxia for 35 days and tamoxifen (1 mg/day) was or was not administered between hypoxia days 17-21. Vibratome lung sections were stained for ATG7, SMA and nuclei (DAPI). Close-ups of boxed regions are shown below. **C**, Quantification of the percent of distal arteriole SMCs that are ATG7^{+}. n=3 mice (1 male, 2 females) per experimental group, 3-4 arteriole analyzed per mouse. Scale bar, 20 μm.

Figure S15. SMC deletion of *Becn1* decreases distal arteriole SMC proliferation. A, Experimental strategy for (B-D). **B,** *Acta2-CreER^{T2}, ROSA26R$^{(Zs/+)}$* mice carrying *Becn1$^{(flox/flox)}$* or *Becn1$^{(+/+)}$* were exposed to hypoxia for 35 days and tamoxifen (1 mg/day) was administered

between hypoxia days 17-21. Zs^+ cells were isolated by FACS, and the mRNA levels of the pro-proliferation marker Ccna and anti-proliferation markers p21 and p27 were measured by qRT-PCR. n=3 mice (2 males, 1 female) per experimental group. **C,** *Acta2-CreER^{T2}, Becn1$^{(flox/flox)}$* mice were exposed to hypoxia for 35 days, and tamoxifen (1 mg/day) was or was not administered between hypoxia days 17-21. Vibratome lung sections were stained for SMA, Ki67 and nuclei (DAPI). Close-ups of boxed regions are shown below. **D,** Percentage of distal arteriole SMCs expressing Ki67 was quantified. n=4 mice (2 males, 2 females) per experimental group, 3 arterioles analyzed per mouse. Scale bar, 20 µm.

Table S1. Demographics of human donors for lung tissue

Diagnostic Category	Age (Yr)	Sex
Control	41	Female
Control	49	Male
Control	54	Male
Control	56	Female
IPAH	41	Female
IPAH	49	Male
IPAH	54	Male
IPAH	56	Female

Table S2. Demographics of human donors for lung RNA

Diagnostic Category	Age (Yr)	Sex
Control	41	Female
Control	43	Male
Control	49	Male
Control	54	Male
Control	56	Female
Control	60	Female
IPAH	25	Male
IPAH	40	Female
IPAH	44	Female
IPAH	51	Male
IPAH	53	Male
IPAH	56	Female

Table S3. Demographics of human donors for lung protein

Diagnostic Category	Age (Yr)	Sex
Control	28	Female
Control	49	Male
Control	54	Male
Control	56	Female
IPAH	25	Male
IPAH	40	Female
IPAH	51	Male
IPAH	56	Female

Table S4. Primers for qRT-PCR

A. Mus Musculus gene primers

Gene name	Forward primer	Reverse primer
mAtg5	TGTGCTTCGAGATGTGTGGTT	GTCAAATAGCTGACTCTTGGCAA
mAtg7	GTTCGCCCCCTTTAATAGTGC	TGAACTCCAACGTCAAGCGG
mBeclin1	ATGGAGGGGTCTAAGGCGTC	TCCTCTCCTGAGTTAGCCTCT
mBnip3	TCCTGGGTAGAACTGCACTTC	GCTGGGCATCCAACAGTATTT
mHif1a	ACCTTCATCGGAAACTCCAAAG	CTGTTAGGCTGGGAAAAGTTAGG
mHif2a	CTGAGGAAGGAGAAATCCCGT	TGTGTCCGAAGGAAGCTGATG
mMap1lc3b	GACCGCTGTAAGGAGGTGC	CTTGACCAACTCGCTCATGTTA
mPdgfb	GGGTGGGACTTTGGTGTAGAGAAG	GGAACGGATTTTGGAGGTAGTGTC
m18S	CGCCGCTAGAGGTGAAATTC	TTGGCAAATGCTTTCGCTC
mBax	TGAAGACAGGGGCCTTTTTG	AATTCGCCGGAGACACTCG
mPuma	AGCAGCACTTAGAGTCGCC	CCTGGGTAAGGGGAGGAGT
mNoxa	GCAGAGCTACCACCTGAGTTC	CTTTTGCGACTTCCCAGGCA
mApaf1	AGTAATGGGTCCTAAGCATGTTG	GCGATTGGGAAAATCACGTAAAA
mBim	GACAGAACCGCAAGGTAATCC	ACTTGTCACAACTCATGGGTG
mBcl2	GTCGCTACCGTCGTGACTTC	CAGACATGCACCTACCCAGC

B. Homo Sapiens gene primers

Gene name	Forward primer	Reverse primer
hAtg5	AAAGATGTGCTTCGAGATGTGT	CACTTTGTCAGTTACCAACGTCA
hAtg7	CAGTTTGCCCCTTTTAGTAGTGC	CCAGCCGATACTCGTTCAGC
hBeclin1	CCATGCAGGTGAGCTTCGT	GAATCTGCGAGAGACACCATC
hHif1a	GAACGTCGAAAAGAAAAGTCTCG	CCTTATCAAGATGCGAACTCACA
hHif2a	CGGAGGTGTTCTATGAGCTGG	AGCTTGTGTGTTCGCAGGAA
hMap1lc3b	AACATGAGCGAGTTGGTCAAG	GCTCGTAGATGTCCGCGAT
hPdgfb	CTCGATCCGCTCCTTTGATGA	CGTTGGTGCGGTCTATGAG
hUlk1	GGCAAGTTCGAGTTCTCCCG	CGACCTCCAAATCGTGCTTCT
hUlk2	ACAGCAAAGGAATCATCCACAG	TGATGCGAATACCACTGACAC
hUlk3	GAAGGACACTCGTGAAGTGGT	ACAATGTGGGGATGTCGAATG
h18S	TAACGAACGAGACTCTGGCAT	CGGACATCTAAGGGCATCACAG
