# Supplementary material for: Endothelial HIFα/PDGF-B to smooth muscle Beclin1 signaling sustains pathological muscularization in pulmonary hypertension
Source: JCI Insight. 2024 Apr 23;9(10):e162449. doi: 10.1172/jci.insight.162449 (PMC11141934; doi:10.1172/jci.insight.162449)

Full unedited blots for Figure 2B

HIF1-α

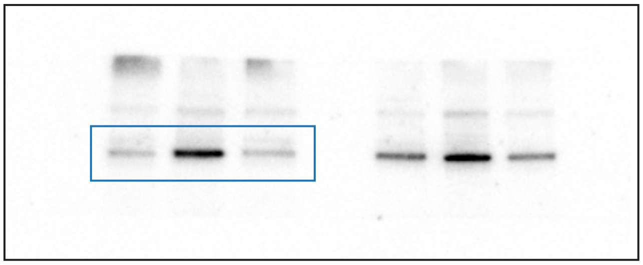

HIF2-α

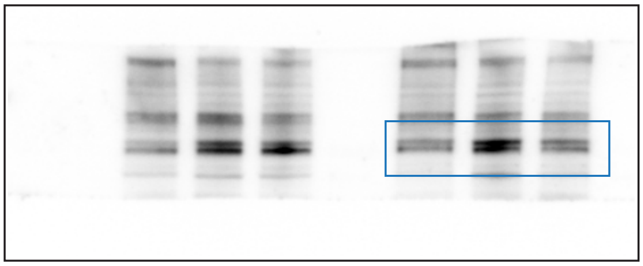

GAPDH

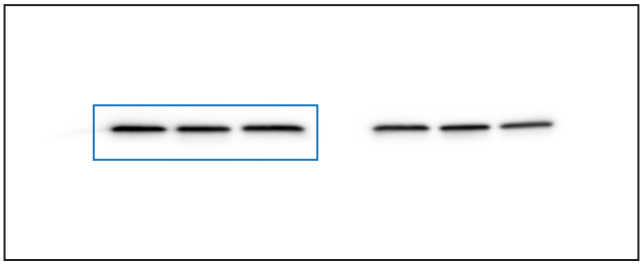

GAPDH

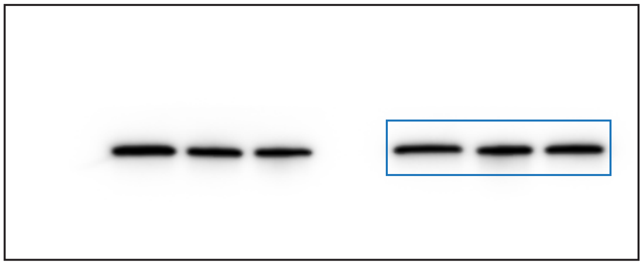

Full unedited blots for Figure 2F

HIF1-α

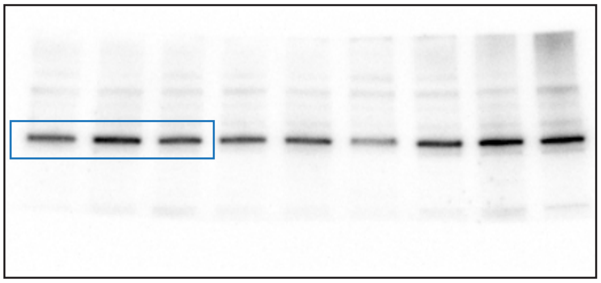

HIF2-α

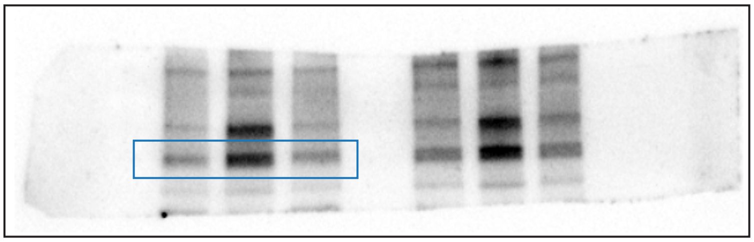

GAPDH

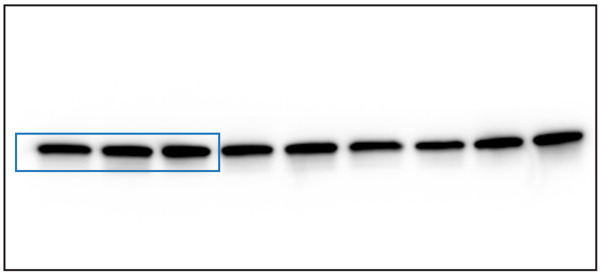

GAPDH

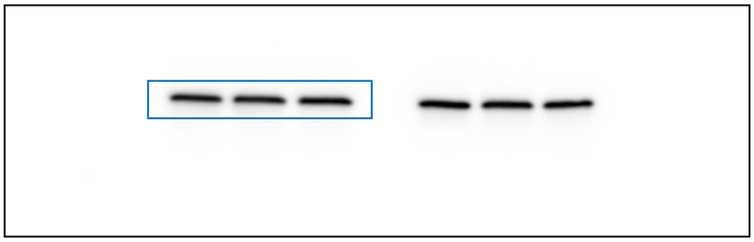

Full unedited blots for Figure 7B

Beclin1

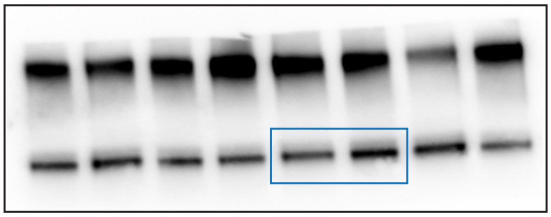

GAPDH

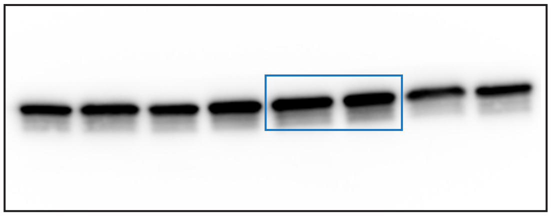

LC3B

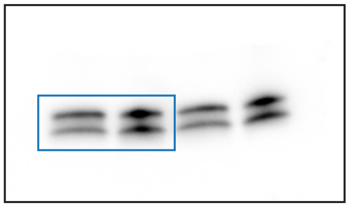

GAPDH

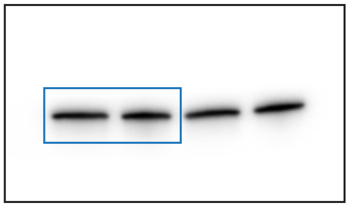

P62

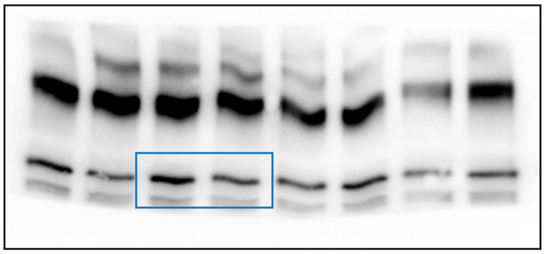

GAPDH

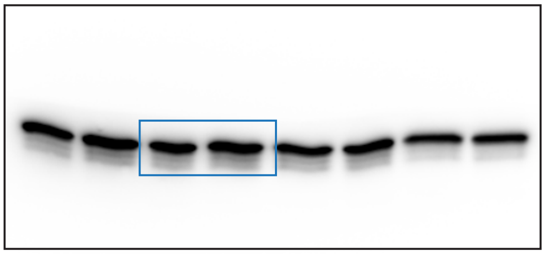

Full unedited blots for Figure S4D

HIF1-α

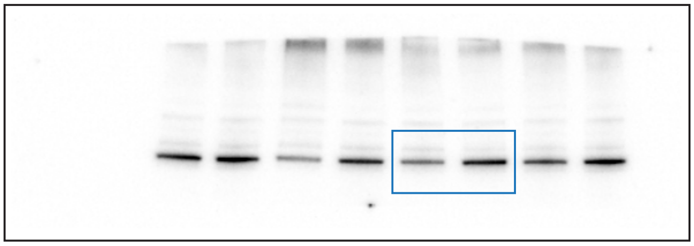

GAPDH

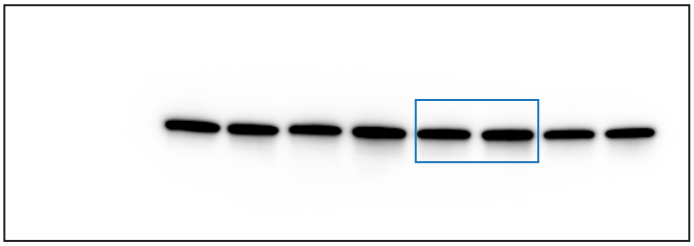

Full unedited blots for Figure S5B

VHL

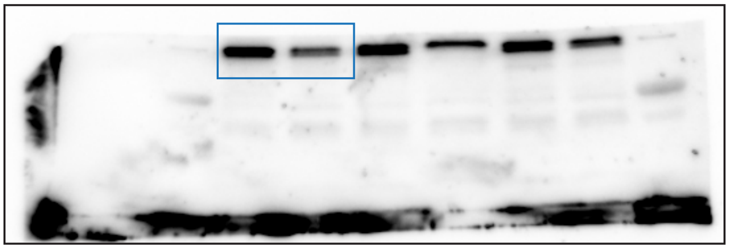

GAPDH

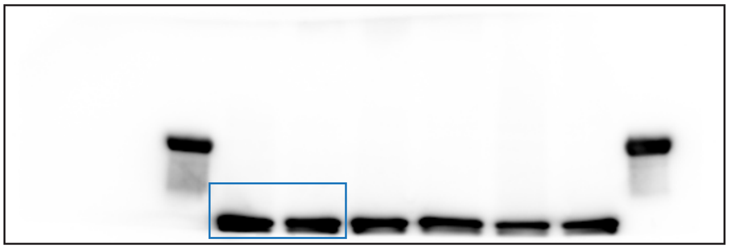

HIF1-α

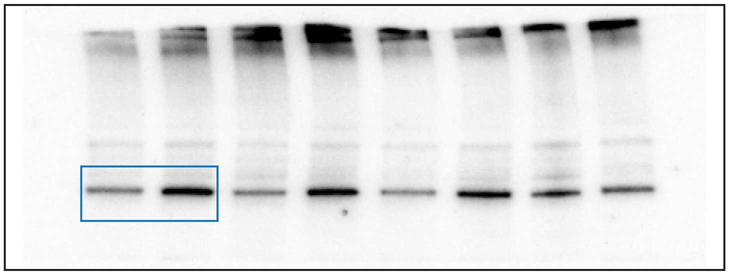

GAPDH

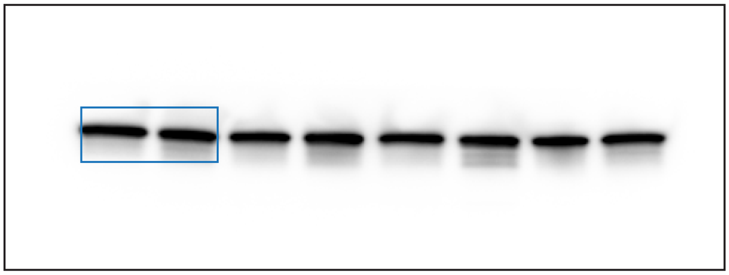

HIF2-α

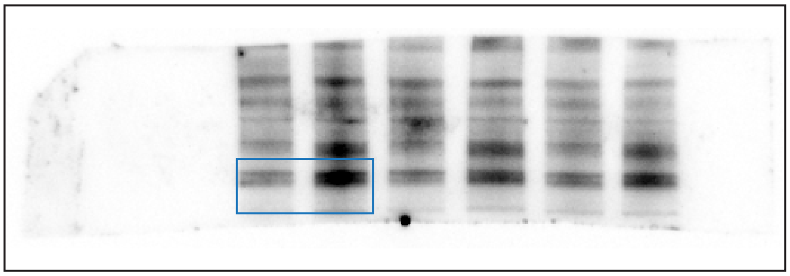

GAPDH

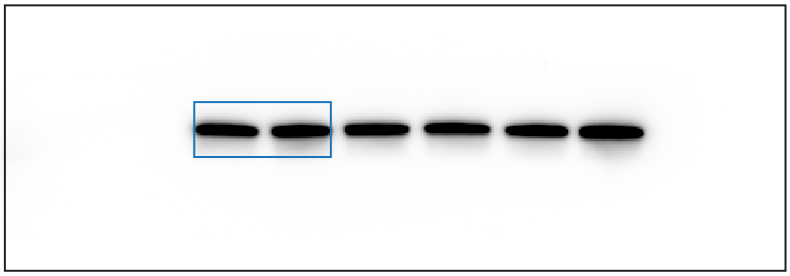

Full unedited blots for Figure S7B

HIF1-α

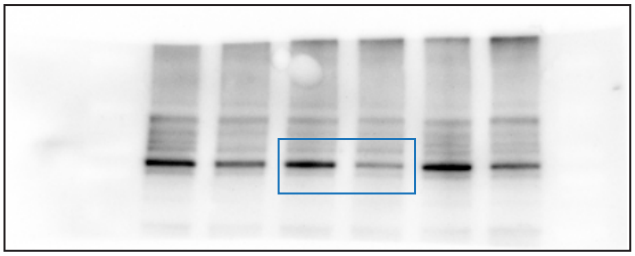

GAPDH

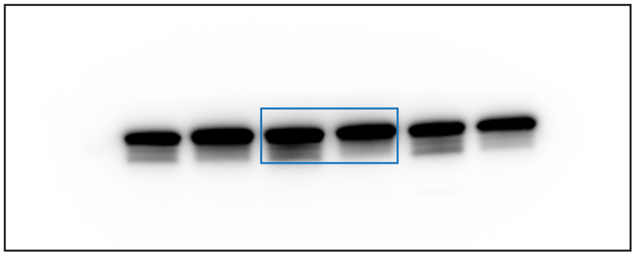

Full unedited blots for Figure S7D

HIF2-α

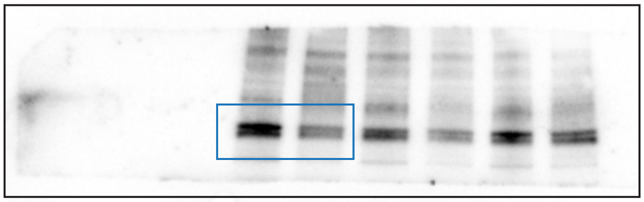

GAPDH

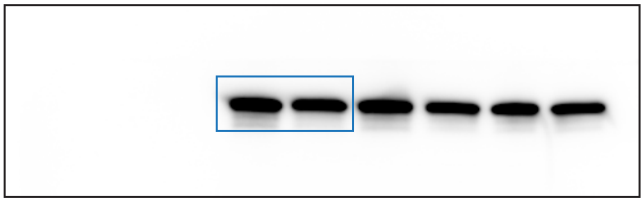

Full unedited blots for Figure S13C

Beclin1

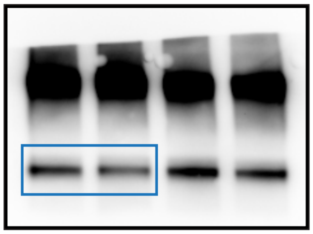

GAPDH

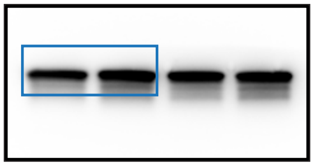

Supplement: Unedited blot and gel images [file jciinsight-9-162449-s059.pdf]
